# Untargeted lipidomics profiling for halal authentication of meatball products from mixed meat sources

**DOI:** 10.1016/j.fochx.2025.102804

**Published:** 2025-07-17

**Authors:** Putri Widyanti Harlina, Asad Nawaz, Raheel Shahzad, Na’Ilah Nur Amalina, Fang Geng, Mohamad Rafi, Vevi Maritha, Aldila Din Pangawikan, Souvia Rahimah, Mohammad Rizwan Khan, Widya Siva Gramita, Adelia Salsabila Nurhasya

**Affiliations:** a Department of Food Industrial Technology, Faculty of Agro-Industrial Technology, Universitas Padjadjaran, 45363 Bandung, Indonesia; b Hunan Engineering Technology Research Center for Comprehensive Development and Utilization of Biomass Resources, College of Chemistry and Bioengineering, Hunan University of Science and Engineering, 425199 Yongzhou, China; c Research Center for Genetic Engineering, National Research and Innovation Agency (BRIN), KST-Cibinong, JI Raya Bogor KM 46, Cibinong 16911, Indonesia; d Meat Processing Key Laboratory of Sichuan Province, School of Food and Biological Engineering, Chengdu University, Chengdu 610106, China; e Department of Chemistry, Faculty of Mathematics and Natural Sciences, IPB University, Jalan Tanjung Kampus IPB Dramaga, Bogor 16680, Indonesia; f Pharmacy Study Program, Faculty of Health and Science, Universitas PGRI, Madiun, Indonesia; g Department of Chemistry, College of Science, King Saud University, P.O. Box 2455, Riyadh 11451, Saudi Arabia

**Keywords:** Halal authentication, Meatball, Lipidomic, Chemometric

## Abstract

Food adulteration with non-halal components, particularly in thermally processed meat products like meatballs, presents significant challenges for conventional DNA and protein-based detection methods. This study applies untargeted lipidomics using ultra-high-performance liquid chromatography–high-resolution mass spectrometry (UHPLC–HRMS) combined with multivariate analysis (principal component analysis [PCA] and partial least squares-discriminant analysis [PLS-DA]) to authenticate halal status in meatball products. Lipid profiles were obtained from beef, goat, pork, and their mixtures, and analyzed under both electrospray ionization in positive mode (ESI+) and electrospray ionization in negative mode (ESI−) modes. Glycerophospholipids emerged as dominant lipid species. PCA and PLS-DA revealed clear separations between halal and non-halal samples, with high model performance (R^2^ = 0.994, Q^2^ = 0.952), demonstrating strong predictive capability. Five key lipid ions—lysophosphatidylethanolamine (LPE)(16:0p) + H, LPE(16:1e) + H, LPE(18:1p) + H, PC(10:0p/23:0) + H, and phosphatidylcholine (PC)(8:0/24:1) + H—were identified as markers for single-source differentiation, while twelve lipids were significant for mixed-meat products. These findings confirm the efficacy and specificity of untargeted lipidomics as a robust method for halal authentication in processed meat products.

## Highlights

• Lipidomics and chemometrics were used to authenticate halal meatball products.
• UHPLC–HRMS successfully detected distinct lipid profiles in single and mixed meat sources (beef, goat, pork).
• PCA and PLS-DA identified specific lipid biomarkers to discriminate halal and non-halal meatball compositions.
• Five lipid markers identified for single-source and twelve for mixed-source products.

## 1 Introduction

### 1.1 Background

Indonesia is an Asian country, with the largest Muslim population in the world (The Royal Islamic Studies Centre, 2023). According to the report of the Royal Islamic Strategic Studies Centre (RISSC) in 2023, there were approximately 237.55 million Muslims in the country, accounting for 86.7 % of the total population (Robi et al., 2023). This demographic reality creates a significant demand for halal-certified food products, emphasizing the need for robust and accurate authentication methods in the Indonesian food industry.

Several studies have shown that halal products are materials declared permissible according to Islamic law (Law of the Republic of Indonesia No. 33 of 2014 on Halal Product Assurance, 2014). The consumption of halal food and beverages is obligatory for Muslims according to Islamic teachings. Consequently, Muslims must know how to select these products, considering their contents and acquisition methods. One of the most widely consumed products by both children and adults is processed meatball (Pamungkas et al., 2014). According to data from the Ministry of Agriculture in 2018, the per capita consumption of meatball in Indonesia reached 31.4 portions per year, with an annual increase of 17.5 %. These products are often produced using various types of meat, including beef, buffalo, pork, goat, and poultry (Sujarwanta et al., 2021). According to the Central Bureau of Statistics of Indonesia (2022), the price of fresh beef in 2022 was Rp134,960 per kg, while goat meat was Rp97,905 per kg in 2021. The increase in prices of these meats led to the risk of economic adulteration, such as adding pork to meatball (Orbayinah et al., 2020).

The illegal addition of pork by producers or suppliers is a form of adulteration (Orbayinah et al., 2020). A notable case of adulteration in Indonesia includes the use of wild boar or pork in Bogor to offset high beef prices (Bempah, 2017). This concern is further supported by reports from the Indonesian Food and Drug Authority (BPOM, 2021) and the Halal Product Assurance Agency (BPJPH, 2022), which document confirmed cases and ongoing regulatory monitoring of meat adulteration practices in Indonesia. Pork addition endangers Muslims, as its consumption is considered haram (forbidden). In addition, adulteration can be harmful due to its minimal impact on product's appearance and texture (Stachniuk et al., 2021; Windarsih et al., 2022). This shows the urgent need for reliable and effective authentication methods.

Authentication methods for processed meat products include protein-based techniques such as Enzyme-Linked Immunosorbent Assay (ELISA), DNA-based techniques like Polymerase Chain Reaction (PCR), and spectroscopic and chromatographic approaches. ELISA is simple and cost-effective but may suffer from false positives and lacks molecular specificity (Uddin, Hossain, Chowdhury, & Johan, 2021; Uddin, Hossain, Sagadevan, et al., 2021; Yang et al., 2018). PCR-based techniques are sensitive and specific; however, thermal processing in meat products like meatballs can degrade DNA, reducing detection accuracy, especially in low-level adulteration (Windarsih et al., 2022). These limitations have driven the exploration of lipidomics as an alternative, as lipids remain more stable under heat and can serve as reliable species-specific markers (Li et al., 2017; Maritha, Harlina, Musfiroh, Muchtaridi, et al., 2023).

Lipidomics is a branch of science that comprehensively studies the function and structure of lipids produced in biological cells, including lipid-lipid, metabolite, and protein interactions (Li et al., 2017). In addition, it is a relatively new method in food analysis, particularly in halal authentication. Lipids are a major component in meat, including beef, goat, or pork. In this study, the characteristics from each source showed different biomarkers, which can be used to identify unwanted lipid sources (Maritha, Harlina, Musfiroh, Muchtaridi, et al., 2023). The advantage of lipidomics analysis is that the lipid profiles can be classified based on the source of the animal species. Ultra High-Performance Liquid Chromatography-High Resolution Mass Spectrometry (UHPLC–HRMS) is the analytical instrument commonly used in lipidomics because it is sensitive and can detect thousands of lipids in meat samples (Maritha, Harlina, Musfiroh, Muchtaridi, et al., 2023; Patterson et al., 2017).

UHPLC–HRMS facilitates sample preparation, data collection, and subsequent stages in lipidomics analysis. In addition, it can record an infinite number of components, making it easier to provide an accurate database (Pérez-Ortega et al., 2016). Mass Spectrometry in this instrument ensures that lipids in the sample can be identified in a single test, making lipidomics with this instrument very efficient. The data produced by UHPLC–HRMS is extensive, requiring chemometric analysis to identify differences in lipids in meat samples. The data obtained can then be presented using various methods, such as PCA and PLS-DA. Several studies have applied lipidomics and chemometric tools for halal authentication, particularly in raw meat samples (Maritha, Harlina, Musfiroh, Muchtaridi, et al., 2023; Maritha, Harlina, Musfiroh, Rafi, et al., 2023; Mokhtar et al., 2023). In contrast, Windarsih et al. (2023) and Stachniuk et al. (2021) have extended these approaches to processed meat products using metabolomics and proteomics. However, few studies have combined untargeted lipidomics with chemometric modeling specifically for thermally processed meatballs—a ready-to-eat product commonly consumed in Indonesia—making the findings more relevant to real-world food authentication challenges. Our study addresses this gap by applying ultra-high-performance liquid chromatography–high-resolution mass spectrometry (UHPLC–HRMS) to enhance detection sensitivity and resolution. The integration of this platform with untargeted lipidomics and multivariate statistical analysis (PCA and PLS-DA) enabled the identification of specific lipid markers that distinguish halal from non-halal meatball products. By emphasizing these aspects, the study highlights its novelty and contributes significantly to the advancement of halal authentication methods for processed food matrices.

## 2 Material and methods

### 2.1 Materials

Beef, pork, goat meat, salt, and tapioca flour were obtained from a local market in Bandung, Indonesia. All meat types were freshly sourced from local Bandung markets within 6 h post-slaughter. The other reagents included sodium sulfate, formic acid, chloroform, methanol, and acetonitrile LC–MS grade, which were all Pro Analysis quality chemical reagents from Merck, Germany.

### 2.2 Methods

#### 2.2.1 Meatball preparation

**Fig. 1:**
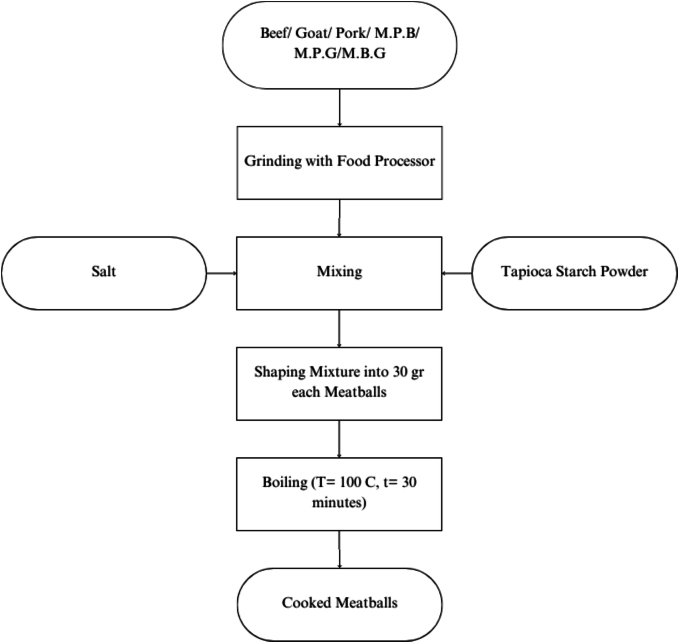
Meatball-making process. MPB: Mixed Pork and Beef; MGP: Mixed Pork and Goat; MBG: Mixed Beef and Goat.

The method for preparing meatball from beef, goat, and pork and their combinations adhered strictly to a modified version of the approach described by Raharjo et al. (2017). Each type of meat was finely ground using a food processor and blended with other ingredients in meat-to-ingredient ratio of 90:10. For combination samples, the ratio of the 2 types of meat was equal. Subsequently, the mixture was shaped into 30-g meatball and boiled for 30 min at 100 °C. Samples were refrigerated at 4 °C and processed within 24 h. Sample integrity was preserved by minimizing freeze-thaw cycles and maintaining chilled conditions throughout preparation. Meatball-making steps were showed in Fig. 1. This experimental study involved 6 groups of meatball samples made from different types of meat with three biological replicates each (total of 18 samples). Meatball sample treatments were listed in Table 1.

**Table 1:** Meatball sample treatment.

Sample Name	Ingredients Ratio (w/w) in gram					Meatball pictures
	Beef	Goat	Pork	Tapioca Flour	Salt	
Beef Meatball	90	0	0	8	2	
Goat Meatball	0	90	0	8	2	
Pork Meatball	0	0	90	8	2	
Pork-Beef Meatball	45	0	45	8	2	
Pork-Goat Meatball	0	45	45	8	2	
Beef-Goat Meatball	45	45	0	8	2	

#### 2.2.2 Lipid extraction from meatball

Lipids were extracted based on the method outlined by Harlina et al. (2021) with slight modifications. Each sample was homogenized in chloroform: methanol solution (120:120:60 v/v/v) at 11,000g for 2 min, followed by sonication for 30 min at 20 °C. The resulting mixture was filtered using a Buchner funnel, and the chloroform phase containing lipids was dried with anhydrous sodium sulfate. Subsequently, the filtrate was concentrated using a rotary evaporator and lipid extracts were stored at −20 °C. The lipid extraction results can be seen in Suppl S1.

#### 2.2.3 Lipidomics analysis

Lipidomic analysis was performed by applying the UHPLC Vanquish and Q Exactive instruments from Thermo Scientific based on the method of Maritha, Harlina, Musfiroh, Muchtaridi, et al. (2023). The column used in UHPLC was Accucore C18 (ThermoScientific) 100 × 2.1 mm, 1.5 μm as the stationary phase. Before injection, 5 mg of the sample was dissolved in 1 mL MeOH and then filtered with a 0.2 μm Nylon membrane. A sample injection of 2 μL was performed at a column temperature of 30 °C and a flow rate of 0.2 mL/min. The mobile phase for this analysis used binary solvents and was divided into 2 types, namely phases A and B. Mobile phase A consisted of 1 mL/L formic acid dissolved in distilled water, while mobile phase B consisted of 1 mL/L formic acid dissolved in acetonitrile. In lipidomic analysis, each mobile phase was delivered in different amounts. Minutes 0 to 1 = 5 mL/L B; 95 mL/L A, 1 to 25 min = 95 mL/ L B; 5 mL/L A, 25 to 28 min = 95 mL/L B; 5 mL/L A, and 28 to 30 min = 5 mL/L A; 5 mL/L B. The Q Exactive conditions for positive and negative ESI were similar except for the spray voltage conditions (3.2 ESI ^+^ for positive ions) and (2.8 ESI^−^ for negative ions). Fragment spectra were obtained at resolutions of 70,000 and 17,500, with a voltage of 3000 V, heat temperature of 320 °C, envelope gas flow of 15 Arb, and additional gas flow of 3 Arb with an *m*/*z* range of 100 to 1500. Lipid analysis and identification were analyzed using LipidSearch 4.1.16 software (Thermo Fisher, CA). Subsequently, the obtained lipid spectra were matched with data in LIPIDMAPS (Lipidomics Database Gateway, (www.lipidmaps.org)), and analyzed using multivariate statistics (PCA). The sample used in this study was repeated thrice.

#### 2.2.4 Statistical and chemometric analysis

Lipidomics analysis was performed in triplicate, and the data obtained from each study were statistically analyzed using 1-way analysis of variance (ANOVA) with Metaboanalyst 6.0 tools. Tests are conducted at a 95 % confidence level (α = 0.05) to determine when the treatments provided significant differences or effects on the test results. When the significance probability value (*p*-value) < 0.05, there was a significant difference, when the *p*-value >0.05, there was no significant difference. Consequently, when significant differences exist, multivariate component analysis (PCA and PLSDA) was performed using Metaboanalyst 6.0 tools, with data normalized via log transformation and auto-scaling. VIP scores >1 were used to identify significant lipid markers. 10-fold cross-validation was applied to validate model robustness.

## 3 Results and discussion

### 3.1 Untargeted lipidomics

Lipidomic LC–MS analysis results for all samples were attached in Supplementary Tables (S2–S7), and the chromatogram results showed absorption followed by MS (Suppl. S8). This absorption showed that the LC–MS conditions were optimal for detecting lipid and sublipid compounds (Suppl. S9). Based on the Total Ion Chromatogram (TIC) generated from LC–MS for beef, goat, pork meatball, and combination meatball, a similar pattern was observed. All LC–MS spectra results were then matched with the Lipidsearch database, yielding over 800 lipid compounds in a single sample. These findings demonstrate the robustness of untargeted lipidomics for profiling complex lipid matrices in processed meat products (Trivedi et al., 2016).

**Fig. 2:**
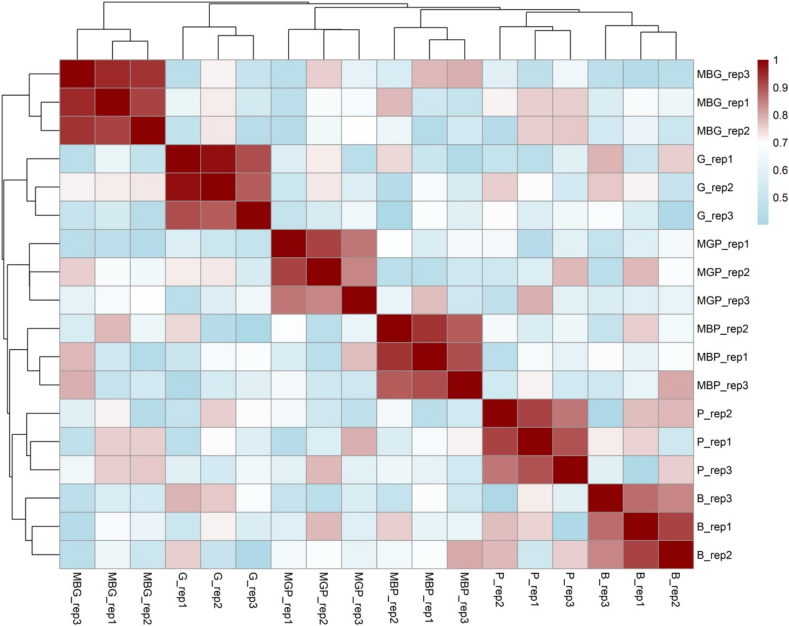
Quality control and pearson correlation analysis between lipidome samples. B: Beef; P: Pork; G: Goat meat; MBP: Mixed Beef Pork; MGP: Mixed Goat Pork; MBG: Mixed Beef Goat.

The lipid analysis results obtained from MS were matched with the Lipidsearch database. In addition, the number of lipid ions detected in the 6 samples in this lipidomic analysis exceeded 800 for each, namely 877 ions for beef, 1019 ions for goat, 1475 ions for pork, 852 ions for pork-beef combination, 1171 ions for pork-goat combination, and 1426 ions for beef-goat combination meatball. This number of lipids showed lipidomics method's ability to accurately select lipid and sublipid compounds, enabling identification of over 800 lipid ions per sample. Maritha, Harlina, Musfiroh, Muchtaridi, et al. (2023) investigated more than 1000 lipid compounds that were detected using lipidomics method. The 6 samples yielded different numbers of compounds, showing that each type of meat had a distinct lipid profile. This study included the selection of 18 samples, which were categorized into 6 groups, each consisting of 3 biological replicates (Fig. 2). The PCA model showed strong reproducibility across samples, with R^2^ values ranging from 0.85 to 1.00. Pearson correlation analysis supported the separation of variables, which were further reduced into uncorrelated principal components for improved group classification. This was executed to enable subsequent chemometric analysis. Previous studies used chemometrics to authenticate halal meat and its derivatives (Harlina et al., 2024; Maritha, Harlina, Musfiroh, Rafi, et al., 2023).

Lipid significance was showed by the 1-way ANOVA results, showing *p*-values <0.05 at a 95 % confidence level. In ESI^−^, the significant lipids were 9 beef meatball, 6 goat meatball, 8 pork meatball, 2 pork-beef combination meatball, 2 pork-goat combination meatball, and 1 beef-goat combination meatball. Meanwhile, in ESI+, the significant lipids were 36 beef meatball, 38 goat meatball, 31 pork meatball, 92 pork-beef combination meatball, 63 pork-goat combination meatball, and 56 beef-goat combination meatball (Table 2). The significant lipid ions from single meatball and combination meatball were presented in supplementary tables.

**Table 2:** Summary of significant lipids in six metball samples.

No	Sample	Lipid in ESI−		Lipid in ESI+	
		Lipid Detected	Significant Lipid	Lipid Detected	Significant Lipid
1	Beef Meatball	182	9	695	36
2	Goat Meatball	108	6	911	38
3	Pork Meatball	144	8	1.331	31
4	Pork-Beef Meatball	86	2	766	92
5	Pork-Goat Meatball	88	2	1.083	63
6	Beef-Goat Meatball	173	1	1.253	56

The highest number of lipids was detected in pork meatball, more than in beef and goat meatball in both ESI+ and ESI−. This was because the type of lipids in pork was more diverse than in beef (Maritha, Harlina, Musfiroh, Muchtaridi, et al., 2023; Trivedi et al., 2016). Compared to lipidomics results for raw beef, pork, and goat meat carried out by Jia et al. (2021) and Maritha, Harlina, Musfiroh, Muchtaridi, et al. (2023), the number of lipid ions in cooked meatball decreased. Previous studies explored the impact of cooking on Tan sheep meat's lipid profile. This showed that boiling reduced over half of the molecules in the lipid class (Jia et al., 2021). Based on the untargeted lipidomics results, more lipids were detected in ESI+ than ESI− across all samples. This difference in ions was due to their polarity factors.

The lipid class category produced in lipidomic analysis, both ESI+ and ESI−, for the 6 samples were glycerophospholipids (GP), which was a major lipid category found in animals. This played a critical role in the composition of membrane phospholipids (Cui et al., 2024; Peter Slotte, 2013). The composition of GP in beef, goat, and pork showed significant differences (Cui et al., 2024). The results of this untargeted lipidomic study differed from those on raw meat from the same parts by Maritha, Harlina, Musfiroh, Muchtaridi, et al. (2023). Where ESI−, the lipid categories fatty acyls, glycerolipids, GP, and sphingolipids were found in beef, and the lipid categories fatty acyls, GP, and sphingolipids were found in pork. In ESI+, the lipid categories GP, glycerolipids, and sphingolipids were observed in raw beef and pork. These differences were due to the cooking process experienced by meatball. Lipid degradation and oxidation occurred during cooking, which reduced the number of sphingolipids and other lipid categories (Li et al., 2022). In addition, reduced lipid content in cooked meatball was associated with water retention during boiling (Semedo Tavares et al., 2018).

GP in ESI− for beef, goat, and pork meatball exhibited specific differences. For significant lipids, the subcategories observed in ESI− were Lyso-phosphatidylethanolamine (LPE) and Phosphatidic Acid (PA). LPE(16:1)-H, PA(29:4/19:2)-H, and PA(30:6/18:0)-H were lipid ions found only in beef meatball. Despite the potential, LPE(16:0)-H was not detected in beef meatball. LPE(17:2)-H was found only in pork meatball, and PA(31:3/17:3)-H was absent in goat meatball but present in both beef and pork meatball. In ESI−, PA(31:17:3)-H and PA(30:6/18:0)-H were the lipids with the highest composition in beef meatball. However, LPE(18:2)-H was the lipid with the highest composition in goat meatball, and LPE(15:1)-H exhibited the highest composition in pork meatball. In combination meatball, only 2 significant lipid ions remained, namely LPE(15:1)-H and LPE(17:2)-H. LPE(17:2)-H was not detected in beef-goat combination meatball.

The GP subcategories detected in ESI+ for single meatball were Lyso-phosphatidylcholine (LPC), LPE, Phosphatidylcholine (PC), Phosphatidylethanolamine (PE), Phosphatidylglycerol (PG), and Phosphatidylinositol (PI). LPC(20:1) + H was a type of LPC found only in beef meatball. LPC(16:1) + H was found only in beef and goat meatball. Meanwhile, LPC(15:0) + H, LPC(16:0e) + H, and LPC(20:0) + H were found only in pork meatball. In the LPE class, LPE(22:6) + H was found only in beef and goat meatball, while LPE(18:1p) + H was found only in pork meatball. A total of 9 PC subcategory lipids were observed only in pork meatball, with the largest composition being PC(18:1e/15:0) + H. PC(10:0p/23:0) + H and PC(8:0/24:1) + H were detected only in goat and pork meatball. PC(4:0/15:2) + H was observed in beef and pork meatball only. In the PC class, only PC(8:0p/8:0) + H was not detected in pork meatball. The PE subcategory contained the highest number of significant lipids. Approximately 25 significant PE subcategory lipids were found in single meatball, with 16 not detected in pork meatball, including PE(12:0/23:1), PE(15:0/20:1) + H, PE(15:1/20:0) + H, PE(16:0e/20:1) + H, PE(16:0p/20:0) + H, PE(16:1/19:0) + H, PE(16:1e/20:0) + H, PE(17:0/18:1) + H, PE(17:1/18:0) + H, PE(18:0e/18:1) + H, PE(18:0e/21:0) + H, PE(18:0p/18:0) + H, PE(19:1/16:0) + H, PE(20:0e/16:1) + H, PE(20:0p/16:0) + H, and PE(20:1e/16:0) + H. The GP subcategories found only in goat meatball were PG and PI, as these were only minimally present in meat (Cui et al., 2024). PE(10:0p/8:0) + H was the lipid with the highest composition in beef and goat meatball, while LPE(16:0p) + H had the highest composition in pork meatball.

A total of 6 GP subclasses were detected, and significant in ESI+ combination meatball samples were LPE, PC, PE, PG, PI, and Phosphatidylserine (PS). LPE(18:0) + H and LPE(18:1p) + H were detected only in pork-beef combination meatball and pork-goat combination meatball. The lipid PC(4:0/15:2) + H was the only lipid found exclusively in pork-beef combination meatball. However, the lipid PC(6:0/11:0) was detected only in pork-beef combination meatball and beef-goat combination meatball. The PE subclass contained the most significant lipids, with 77 ions. Of these, 22 were found only in pork-beef combination meatball, with the largest composition in PE(14:1/17:2) + H. A total of 2 lipids, PG(8:0/20:4) + H and PG(8:0p/24:7) + H, were detected only in pork-beef combination meatball. The PI subclass was the only lipid subclass not detected in beef-goat combination meatball. PI(19:1/19:4) + H was detected only in pork-beef combination meatball, while PI(24:0/22:5) + H was found in samples containing pork. The lipid PE(8:0p/15:2) + H had the largest composition in pork-beef combination meatball, while PE(4:0/19:5) + H exhibited the largest composition in pork-goat and beef-goat combination meatball. The summary of significant lipid numbers was presented in Table 2.

**Fig. 3:**
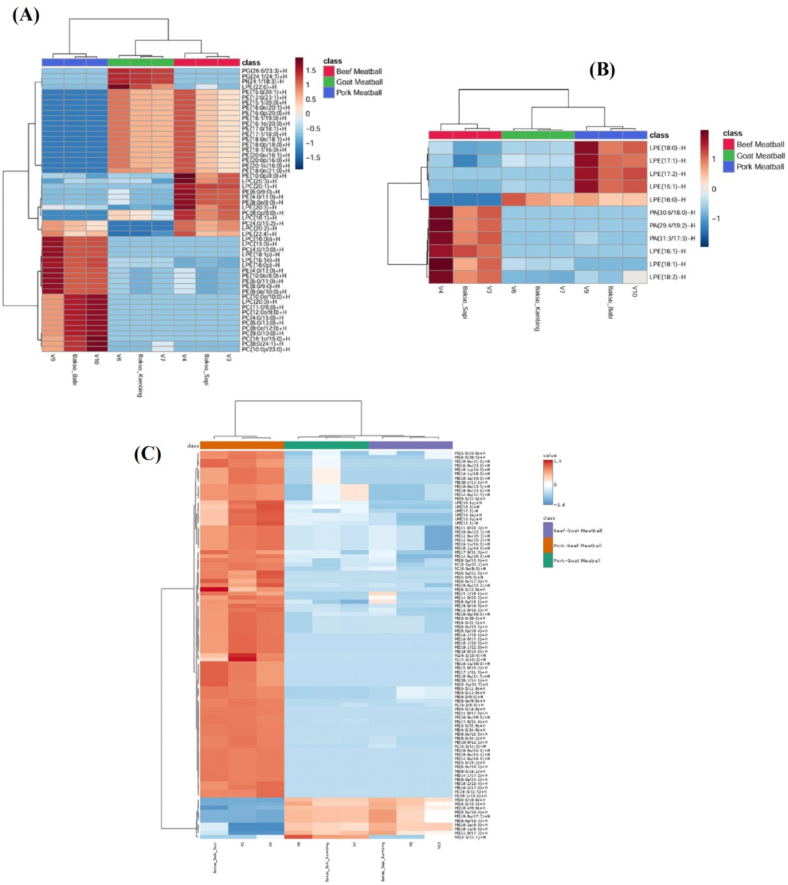
Heatmap clustering of single meatball in (A) ESI positive; (B) ESI negative and (C) heatmap clustering of combination meatball.

Heatmap clustering could present a more precise overview of the lipid profiles in the 6 meatball samples, showing their variation among meatball samples. This visualization identified differences and similarities in lipid composition between samples. Heatmap clustering for single and combination meatball was presented in Fig. 3. In Fig. 3A, beef, goat, and pork meatballs analyzed in ESI+ mode formed distinct clusters, reflecting their compositional differences. Pork meatball samples showed high expression of specific lipid groups, while goat and beef samples exhibited closer profiles.

Fig. 3B showed the lipid profiles of beef, goat, and pork meatball in ESI−. These samples formed separate clusters, showing different lipid profiles for each meatball type. According to the heatmap, pork meatball exhibited high expression of lipids in the upper part. However, beef meatball showed higher lipid expression in the lower part compared to the other 2. The heatmap showed that pork and goat meatball had more similar lipid profiles compared to beef meatball in ESI−.

In Fig. 3C, the lipid profiles of the meat type and combinations formed separate clusters, showing different lipid profiles for each meatball type. According to the heatmap, pork-beef combination meatball exhibited high lipid expression levels in most lipid profiles. However, pork-goat combination meatball and beef-goat combination meatball had similar lipid expression patterns. This heatmap showed that pork-goat and beef-goat combination meatball had closer lipid profiles compared to pork-beef combination meatball. These 3 heatmap clusters could provide insights into lipid composition among samples (Cui et al., 2024; Maritha, Harlina, Musfiroh, Muchtaridi, et al., 2023).

### 3.2 Chemometric lipidomics

Lipidomic analysis generated extensive, multidimensional data, requiring robust data processing to enable meaningful interpretation. In this study, chemometric tools were applied to reduce dimensionality and extract relevant patterns from the complex lipidomic dataset. All variables were analyzed collectively using covariance and correlation (Saccenti et al., 2014), allowing the detection of significant differences in lipid profiles among beef, goat, pork, and combination meatball samples. PCA was used to explore grouping patterns and visualize lipid profile variations, while PLS-DA identified the lipid species most relevant for class separation (Böhme et al., 2019; Righetti et al., 2018; Wu et al., 2020).

The role of chemometrics was critical to the study's aim of halal authentication, as PCA and PLS-DA facilitated the distinction between halal and non-halal meatball samples. This multivariate approach revealed five lipid markers that effectively distinguished single meat sources and twelve additional markers for mixed meat products. The model exhibited excellent classification performance (R^2^ = 0.994, Q^2^ = 0.952), confirming the reliability of the identified markers. Thus, chemometric analysis was not only essential for managing the data's complexity but also contributed directly to the successful identification of specific lipid biomarkers for pork adulteration in halal meatball products.

**Fig. 4:**
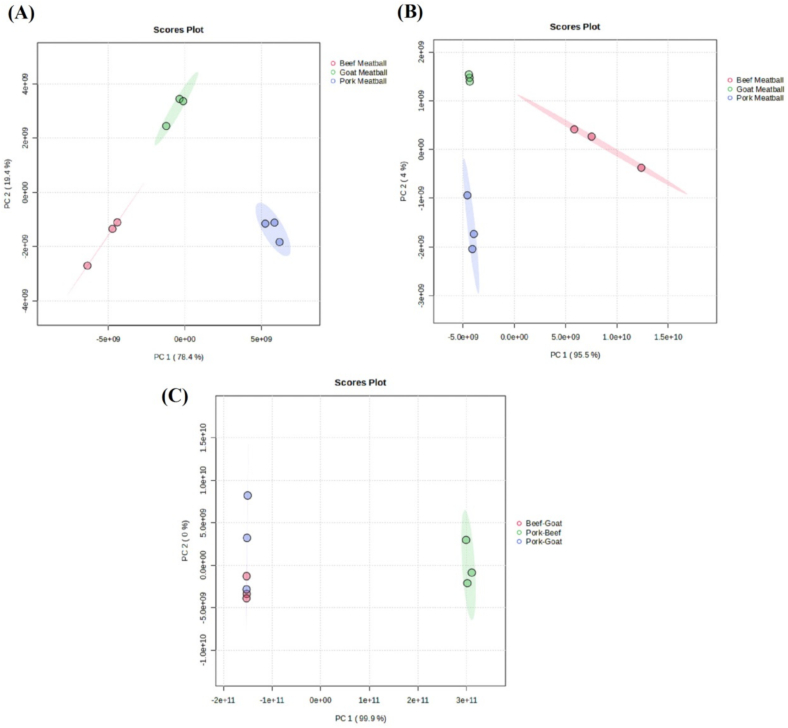
PCA of beef, goat, and pork meatball in (A) positive ESI; (B) negative ESI and (C) PCA of beef-pork, beef-goat, and goat-pork combination meatball.

Fig. 4A showed the PCA results for the lipid profiles of beef, goat, and pork meatball in ESI+. PC 1 described 78.4 % of the total variance, while PC 2 explained 19.4 % of the total variance. The PCA plot showed 3 distinct groups, including red circles representing beef meatball, blue circles representing pork meatball, and green circles representing goat meatball. Therefore, the formation of these groups showed that beef, goat, and pork meatball had different lipid profiles in positive ESI.

Fig. 4B showed the PCA results for the lipid profiles of beef, goat, and pork meatball in ESI−. PC 1 explained 95.5 % of the total variance, while PC 2 described 4 % of the total variance. The PCA plot showed 3 distinct groups, namely red circles representing beef meatball, blue circles representing pork meatball, and green circles representing goat meatball. The formation of these groups showed that beef, goat, and pork meatball also had different lipid profiles in negative ion mode. Therefore, PCA resulted in both ESI modes showing that beef, goat, and pork lipid compositions and contents were significantly different (Cui et al., 2024).

In Fig. 4C, the PCA results for the lipid profiles of beef-pork, beef-goat, and goat-pork combination meatball in both ESI modes were showed. Unlike the PCA for individual meatball, there was only 1 PCA plot for combination meatball because there were 2 significant lipid variables in negative ion mode. PC 1 explained 99.9 % of the total variance, while PC 2 described 0 % of the total variance. The PCA plot showed 3 distinct groups, namely blue circles representing the pork-goat combination, green circles representing the pork-beef combination, and red circles representing the beef-goat combination. In addition, the proximity of the beef-goat and goat-pork combinations suggested a slight similarity between these 2 samples. The formation of these groups showed that beef, goat, and pork had different lipid profiles.

Based on PCA Figures, it could be concluded that lipid profiles in ESI+ and ESI− were used as references for halal authentication. However, further PLS-DA testing was required to identify which lipid ions most significantly influenced halal determination (Böhme et al., 2019). PLS-DA combined PLS with discriminant analysis, improving discrimination and classification capabilities (Jiménez-Carvelo et al., 2021). Identifying lipids crucial for halal authentication could be determined through Variable Importance in Projection (VIP) scores exceeding 1, predominantly found in pork and pork combination meatball, as showed by the heatmap legend colors. The results of PLS-DA for single and combination meatball were presented in Fig. 4.

**Fig. 5:**
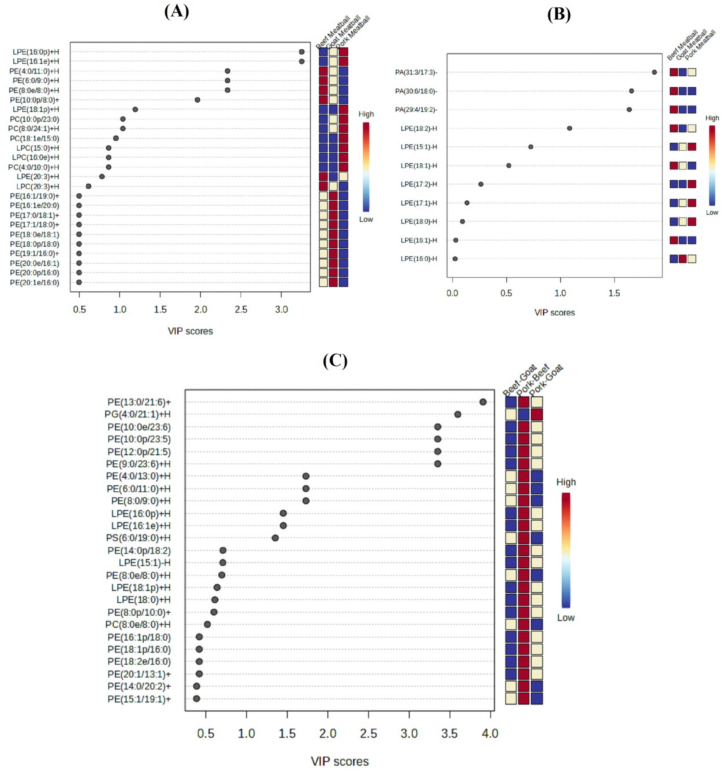
VIP score of single meatball in (A) positive ESI; (B) negative ESI; and (C) VIP score of combination meatball.

Fig. 5A presented the PLS-DA results for the top 25 lipid ions in single meatball in ESI+. According to the VIP scores and the heatmap colors for pork meatball, 5 lipid ions, namely LPE(16:0p) + H, LPE(16:1e) + H, LPE(18:1p) + H, PC(10:0p/23:0) + H, and PC(8:0/24:1) + H could be used for halal authentication of beef, goat, and pork meatball. This PLS-DA model yielded an R^2^ value of 0.994, showing that it explained almost all of the variance in the variables, making it a good model. The Q^2^ value of 0.952 showed that the model had strong predictive capability and could accurately predict new data.

In Fig. 5B, the PLS-DA results for the top 11 lipid ions in single meatball in ESI− were showed. Based on VIP scores and the heatmap colors for pork meatball, no lipid ions in ESI^−^ were used for halal authentication of beef, goat, and pork meatball. Therefore, halal authentication using lipidomics was more effectively performed using lipid profiles in ESI+.

Fig. 5C showed the PLS-DA results for the top 25 lipid ions in combination meatball. Based on VIP scores, 12 lipid ions, namely PE(13:0/21:6) + H, PG(4:0/21:1) + H, PE(10:0e/23:6) + H, PE(10:0p/23:5) + H, PE(12:0p/21:5) + H, PE(9:0/23:6) + H, PE(4:0/13:0) + H, PE(6:0/11:0) + H, PE(8:0/9:0) + H, LPE(16:0p) + H, LPE(16:1e) + H, and PS(6:0/19:0) was used for halal authentication of pork-beef, pork-goat, and beef-goat combination meatball. Subsequently, these lipids were compared with significant lipids from single meatball. The lipids PE(4:0/13:0) + H, PE(6:0/11:0) + H, PE(8:0/9:0) + H, LPE(16:0p) + H, and LPE(16:1e) + H were present in high concentrations in pork meatball, suggesting the presence of pork in those meatball. In addition, 7 other lipid ions were identified but had not been attributed to a specific source, and were not significant in individual meatball samples.

## 4 Conclusions

This study addressed the critical issue of pork adulteration in halal meatball products by applying untargeted lipidomics coupled with multivariate chemometric analysis. Through high-resolution lipid profiling and the use of PCA and PLS-DA, we achieved clear separation between halal and non-halal samples, with strong model performance (R^2^ = 0.994, Q^2^ = 0.952). Five lipid markers were identified as robust indicators for single meat sources, and twelve additional markers were significant for mixed meat products. This is the first study to apply untargeted lipidomics and chemometric modeling to thermally processed meatball products using UHPLC–HRMS, offering high practical relevance. This study successfully identified specific lipid markers for pork adulteration in meatball products, enabling robust halal authentication. Looking ahead, this method holds promise for broader application to other processed meat products and could be integrated into regulatory screening pipelines through targeted lipidomics.

Future studies should also incorporate ROC curves and confusion matrices to assess the performance and reliability of lipid-based markers. Additionally, spike-in validation experiments using known meat mixtures are recommended to confirm the source attribution of lipid markers, particularly those not clearly linked to specific meat types.

## CRediT authorship contribution statement

**Putri Widyanti Harlina:** Writing – review & editing, Writing – original draft, Visualization, Validation, Supervision, Software, Resources, Project administration, Investigation, Funding acquisition, Data curation, Conceptualization. **Asad Nawaz:** Writing – review & editing, Supervision. **Raheel Shahzad:** Writing – review & editing, Validation. **Na’Ilah Nur Amalina:** Writing – original draft, Software, Investigation, Data curation. **Fang Geng:** Writing – review & editing, Validation. **Mohamad Rafi:** Writing – review & editing, Validation. **Vevi Maritha:** Writing – original draft, Software, Investigation, Data curation. **Aldila Din Pangawikan:** Writing – review & editing, Supervision. **Souvia Rahimah:** Writing – review & editing, Supervision. **Mohammad Rizwan Khan:** Writing – review & editing, Validation. **Widya Siva Gramita:** Software, Methodology. **Adelia Salsabila Nurhasya:** Software, Methodology.

## Funding

This study was funded by the Internal Funding of Universitas Padjadjaran (RKKU, 1029/UN6.3.1/PT.00/2025).

## Declaration of competing interest

The authors declare that they have no known competing financial interests or personal relationships that could have appeared to influence the work reported in this paper.

## Data Availability

The data presented in this study are available on request from the corresponding author.

## Appendix A Supplementary data

Supplementary material. Image 7
