# Supplementary material for: Untargeted lipidomics profiling for halal authentication of meatball products from mixed meat sources
Source: Food Chem X. 2025 Jul 17;29:102804. doi: 10.1016/j.fochx.2025.102804 (PMC12311581; doi:10.1016/j.fochx.2025.102804)
Supplement: Supplementary file 1 — Supplementary material. [file mmc1.docx]

**Supplementary Materials**

**Table S1.** Lipid Extraction Results

| No | Sample | Meatball Picture | Extraction Volume (mL) | Extract Picture |
| --- | --- | --- | --- | --- |
| 1. | Beef Meatball | 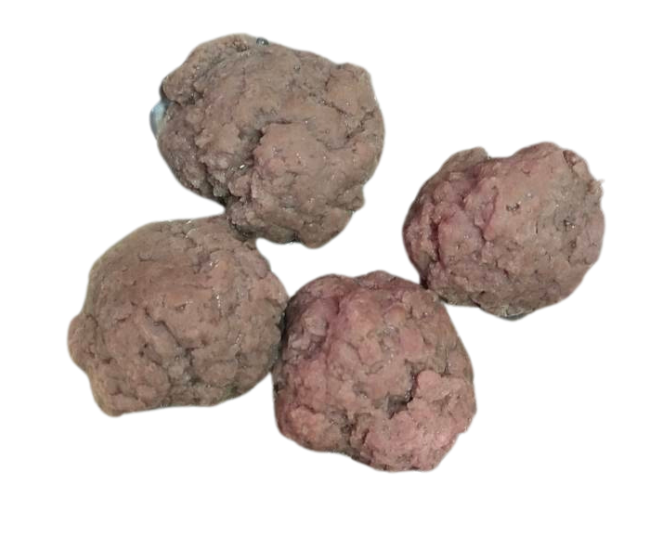 | 2,7 | 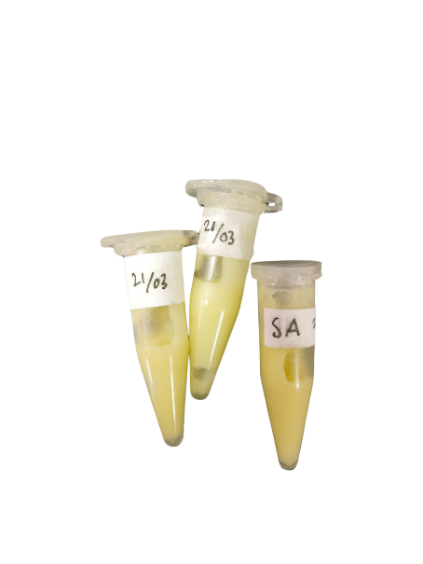 |
| 2. | Goat Meatball | 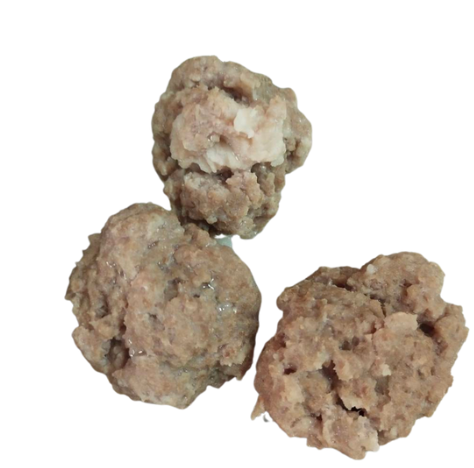 | 4,5 | 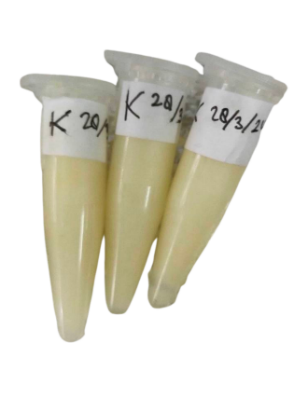 |
| 3. | Pork Meatball | 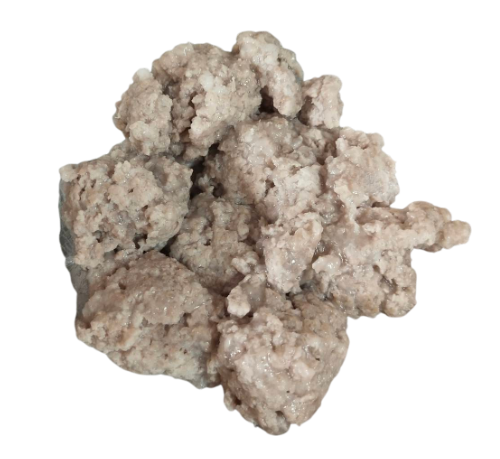 | 2,0 | 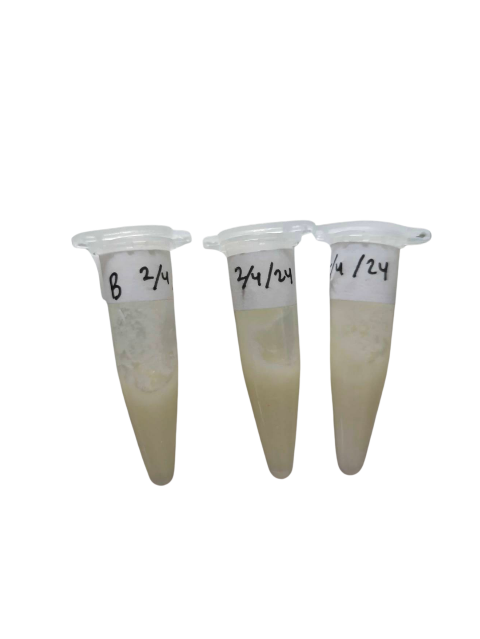 |
| 4. | Pork-Beef Meatball | 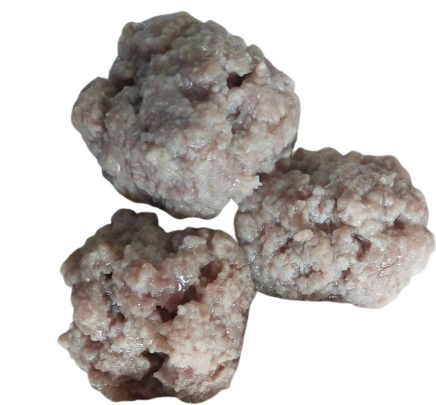 | 3,0 | 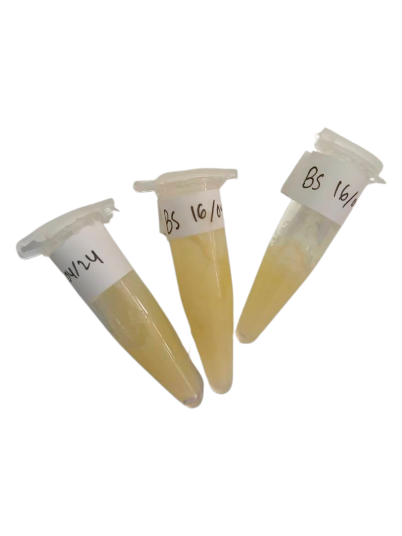 |
| 5. | Pork-Goat Meatball | 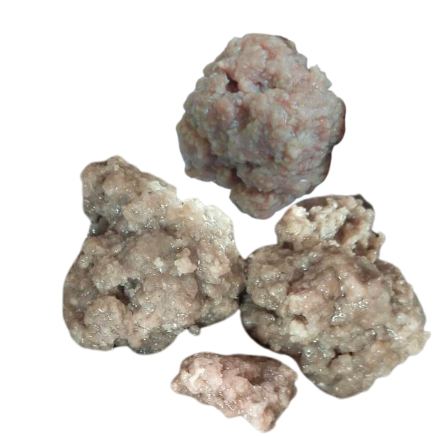 | 3,2 | 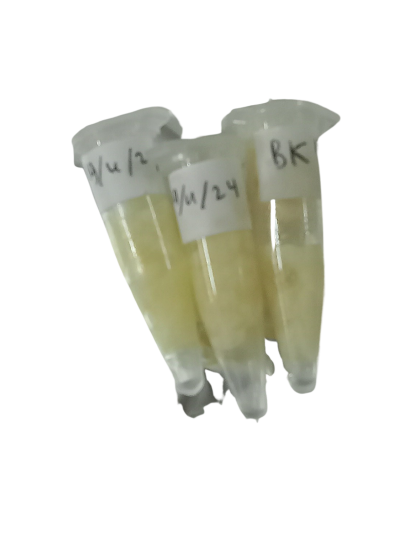 |
| 6. | Beef-Goat Meatball | 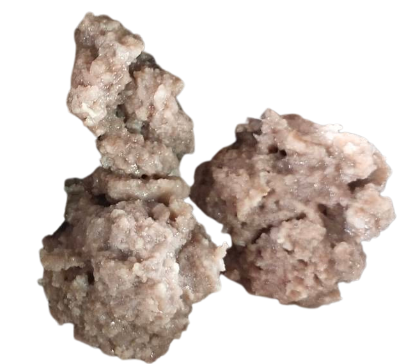 | 2,5 | 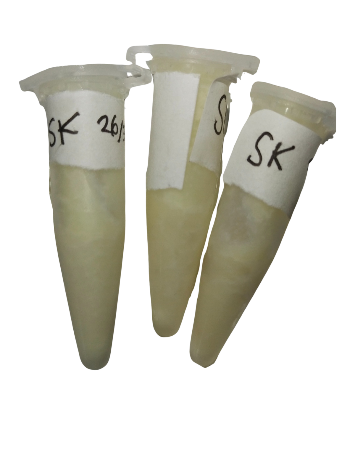 |

**Table S2.** Significant Lipid Results for Beef Meatballs

| LipidIon | Class | Formula | Rt | CalcMz | X Area |
| --- | --- | --- | --- | --- | --- |
| LPC(16:1)+H | LPC | C24 H49 O7 N1 P1 | 17,752684 | 494,3241185 | 4,60E+06 |
| LPC(20:1)+H | LPC | C28 H57 O7 N1 P1 | 21,417901 | 550,3867185 | 5,78E+06 |
| LPC(20:2)+H | LPC | C28 H55 O7 N1 P1 | 19,804049 | 548,3710685 | 5,19E+06 |
| LPC(20:3)+H | LPC | C28 H53 O7 N1 P1 | 19,41844 | 546,3554185 | 1,09E+07 |
| LPE(16:0p)+H | LPE | C21 H45 O6 N1 P1 | 20,336258 | 438,2979035 | 1,12E+06 |
| LPE(16:1e)+H | LPE | C21 H45 O6 N1 P1 | 20,336258 | 438,2979035 | 1,12E+06 |
| LPE(20:3)+H | LPE | C25 H47 O7 N1 P1 | 19,203352 | 504,3084685 | 2,23E+07 |
| LPE(22:4)+H | LPE | C27 H49 O7 N1 P1 | 20,123031 | 530,3241185 | 8,59E+06 |
| LPE(22:6)+H | LPE | C27 H45 O7 N1 P1 | 18,463983 | 526,2928185 | 4,55E+05 |
| PC(4:0/15:2)+H | PC | C27 H51 O8 N1 P1 | 19,804049 | 548,3346835 | 5,19E+06 |
| PC(8:0p/8:0)+H | PC | C24 H49 O7 N1 P1 | 17,752684 | 494,3241185 | 4,60E+06 |
| PE(10:0e/8:0)+H | PE | C23 H49 O7 N1 P1 | 22,152021 | 482,3241185 | 4,36E+06 |
| PE(10:0p/8:0)+H | PE | C23 H47 O7 N1 P1 | 19,90921 | 480,3084685 | 3,92E+07 |
| PE(12:0/23:1)+H | PE | C40 H79 O8 N1 P1 | 22,638678 | 732,5537835 | 8,85E+06 |
| PE(15:0/20:1)+H | PE | C40 H79 O8 N1 P1 | 22,638678 | 732,5537835 | 8,85E+06 |
| PE(15:1/20:0)+H | PE | C40 H79 O8 N1 P1 | 22,638678 | 732,5537835 | 8,85E+06 |
| PE(16:0e/20:1)+H | PE | C41 H83 O7 N1 P1 | 22,638678 | 732,5901685 | 8,85E+06 |
| PE(16:0p/20:0)+H | PE | C41 H83 O7 N1 P1 | 22,638678 | 732,5901685 | 8,85E+06 |
| PE(16:1/19:0)+H | PE | C40 H79 O8 N1 P1 | 22,638678 | 732,5537835 | 8,85E+06 |
| PE(16:1e/20:0)+H | PE | C41 H83 O7 N1 P1 | 22,638678 | 732,5901685 | 8,85E+06 |
| PE(17:0/18:1)+H | PE | C40 H79 O8 N1 P1 | 22,638678 | 732,5537835 | 8,85E+06 |
| PE(17:1/18:0)+H | PE | C40 H79 O8 N1 P1 | 22,638678 | 732,5537835 | 8,85E+06 |
| PE(18:0e/18:1)+H | PE | C41 H83 O7 N1 P1 | 22,638678 | 732,5901685 | 8,85E+06 |
| PE(18:0e/21:0)+H | PE | C44 H91 O7 N1 P1 | 22,603246 | 776,6527685 | 5,92E+06 |
| PE(18:0p/18:0)+H | PE | C41 H83 O7 N1 P1 | 22,638678 | 732,5901685 | 8,85E+06 |
| PE(19:1/16:0)+H | PE | C40 H79 O8 N1 P1 | 22,638678 | 732,5537835 | 8,85E+06 |
| PE(20:0e/16:1)+H | PE | C41 H83 O7 N1 P1 | 22,638678 | 732,5901685 | 8,85E+06 |
| PE(20:0p/16:0)+H | PE | C41 H83 O7 N1 P1 | 22,638678 | 732,5901685 | 8,85E+06 |
| PE(20:1e/16:0)+H | PE | C41 H83 O7 N1 P1 | 22,638678 | 732,5901685 | 8,85E+06 |
| PE(4:0/11:0)+H | PE | C20 H41 O8 N1 P1 | 23,091916 | 454,2564335 | 3,67E+07 |
| PE(4:0/13:0)+H | PE | C22 H45 O8 N1 P1 | 22,152021 | 482,2877335 | 4,36E+06 |
| PE(6:0/11:0)+H | PE | C22 H45 O8 N1 P1 | 22,152021 | 482,2877335 | 4,36E+06 |
| PE(6:0/9:0)+H | PE | C20 H41 O8 N1 P1 | 23,091916 | 454,2564335 | 3,67E+07 |
| PE(8:0/9:0)+H | PE | C22 H45 O8 N1 P1 | 22,152021 | 482,2877335 | 4,36E+06 |
| PE(8:0e/10:0)+H | PE | C23 H49 O7 N1 P1 | 22,152021 | 482,3241185 | 4,36E+06 |
| PE(8:0e/8:0)+H | PE | C21 H45 O7 N1 P1 | 23,091916 | 454,2928185 | 3,67E+07 |
| LPE(15:1)-H | LPE | C20 H39 O7 N1 P1 | 20,249837 | 436,2469655 | 2,43E+06 |
| LPE(18:2)-H | LPE | C23 H43 O7 N1 P1 | 18,300933 | 476,2782655 | 6,20E+07 |
| LPE(17:1)-H | LPE | C22 H43 O7 N1 P1 | 23,013509 | 464,2782655 | 1,04E+06 |
| LPE(18:0)-H | LPE | C23 H47 O7 N1 P1 | 22,097771 | 480,3095655 | 2,19E+06 |
| LPE(18:1)-H | LPE | C23 H45 O7 N1 P1 | 19,88982 | 478,2939155 | 2,39E+07 |
| PA(31:3/17:3)-H | PA | C51 H88 O8 N0 P1 | 25,977388 | 859,6222315 | 7,70E+07 |
| PA(30:6/18:0)-H | PA | C51 H88 O8 N0 P1 | 24,080117 | 859,6222315 | 7,70E+07 |
| PA(29:4/19:2)-H | PA | C51 H88 O8 N0 P1 | 24,080117 | 859,6222315 | 7,61E+07 |
| LPE(16:1)-H | LPE | C21 H41 O7 N1 P1 | 17,556282 | 450,2626155 | 1,46E+06 |

**Table S3.** Significant Lipid Results for Goat Meatballs

| **LipidIon** | **Class** | **Formula** | **Rt** | **CalcMz** | **X Area** |
| --- | --- | --- | --- | --- | --- |
| LPC(16:1)+H | LPC | C24 H49 O7 N1 P1 | 17,808606 | 494,32412 | 2,64E+06 |
| LPC(20:3)+H | LPC | C28 H53 O7 N1 P1 | 19,478896 | 546,35542 | 2,08E+06 |
| LPE(16:0p)+H | LPE | C21 H45 O6 N1 P1 | 20,291578 | 438,2979 | 9,43E+06 |
| LPE(16:1e)+H | LPE | C21 H45 O6 N1 P1 | 20,291578 | 438,2979 | 9,43E+06 |
| LPE(20:3)+H | LPE | C25 H47 O7 N1 P1 | 19,234264 | 504,30847 | 5,63E+06 |
| LPE(22:4)+H | LPE | C27 H49 O7 N1 P1 | 20,113614 | 530,32412 | 5,19E+05 |
| LPE(22:6)+H | LPE | C27 H45 O7 N1 P1 | 18,483453 | 526,29282 | 4,05E+06 |
| PC(8:0p/8:0)+H | PC | C24 H49 O7 N1 P1 | 17,808606 | 494,32412 | 2,64E+06 |
| PE(10:0e/8:0)+H | PE | C23 H49 O7 N1 P1 | 22,113699 | 482,32412 | 4,14E+06 |
| PE(10:0p/8:0)+H | PE | C23 H47 O7 N1 P1 | 20,15323 | 480,30847 | 1,15E+07 |
| PE(12:0/23:1)+H | PE | C40 H79 O8 N1 P1 | 22,702617 | 732,55378 | 9,15E+06 |
| PE(15:0/20:1)+H | PE | C40 H79 O8 N1 P1 | 22,702617 | 732,55378 | 9,15E+06 |
| PE(15:1/20:0)+H | PE | C40 H79 O8 N1 P1 | 22,702617 | 732,55378 | 9,15E+06 |
| PE(16:0e/20:1)+H | PE | C41 H83 O7 N1 P1 | 22,702617 | 732,59017 | 9,15E+06 |
| PE(16:0p/20:0)+H | PE | C41 H83 O7 N1 P1 | 22,702617 | 732,59017 | 9,15E+06 |
| PE(16:1/19:0)+H | PE | C40 H79 O8 N1 P1 | 22,702617 | 732,55378 | 9,15E+06 |
| PE(16:1e/20:0)+H | PE | C41 H83 O7 N1 P1 | 22,702617 | 732,59017 | 9,15E+06 |
| PE(17:0/18:1)+H | PE | C40 H79 O8 N1 P1 | 22,702617 | 732,55378 | 9,15E+06 |
| PE(17:1/18:0)+H | PE | C40 H79 O8 N1 P1 | 22,702617 | 732,55378 | 9,15E+06 |
| PE(18:0e/18:1)+H | PE | C41 H83 O7 N1 P1 | 22,702617 | 732,59017 | 9,15E+06 |
| PE(18:0e/21:0)+H | PE | C44 H91 O7 N1 P1 | 22,632633 | 776,65277 | 6,12E+06 |
| PE(18:0p/18:0)+H | PE | C41 H83 O7 N1 P1 | 22,702617 | 732,59017 | 9,15E+06 |
| PE(19:1/16:0)+H | PE | C40 H79 O8 N1 P1 | 22,702617 | 732,55378 | 9,15E+06 |
| PE(20:0e/16:1)+H | PE | C41 H83 O7 N1 P1 | 22,702617 | 732,59017 | 9,15E+06 |
| PE(20:0p/16:0)+H | PE | C41 H83 O7 N1 P1 | 22,702617 | 732,59017 | 9,15E+06 |
| PE(20:1e/16:0)+H | PE | C41 H83 O7 N1 P1 | 22,702617 | 732,59017 | 9,15E+06 |
| PE(4:0/11:0)+H | PE | C20 H41 O8 N1 P1 | 19,127343 | 454,25643 | 4,87E+06 |
| PE(4:0/13:0)+H | PE | C22 H45 O8 N1 P1 | 22,113699 | 482,28773 | 4,14E+06 |
| PE(6:0/11:0)+H | PE | C22 H45 O8 N1 P1 | 22,113699 | 482,28773 | 4,14E+06 |
| PE(6:0/9:0)+H | PE | C20 H41 O8 N1 P1 | 19,127343 | 454,25643 | 4,87E+06 |
| PE(8:0/9:0)+H | PE | C22 H45 O8 N1 P1 | 22,113699 | 482,28773 | 4,14E+06 |
| PE(8:0e/10:0)+H | PE | C23 H49 O7 N1 P1 | 22,113699 | 482,32412 | 4,14E+06 |
| PE(8:0e/8:0)+H | PE | C21 H45 O7 N1 P1 | 19,127343 | 454,29282 | 4,87E+06 |
| LPE(15:1)-H | LPE | C20 H39 O7 N1 P1 | 20,273818 | 436,24697 | 5,01E+06 |
| LPE(18:2)-H | LPE | C23 H43 O7 N1 P1 | 18,682507 | 476,27827 | 8,79E+06 |
| LPE(17:1)-H | LPE | C22 H43 O7 N1 P1 | 23,0021 | 464,27827 | 2,37E+06 |
| LPE(18:0)-H | LPE | C23 H47 O7 N1 P1 | 22,131674 | 480,30957 | 2,58E+06 |
| LPE(18:1)-H | LPE | C23 H45 O7 N1 P1 | 20,168045 | 478,29392 | 7,00E+06 |
| LPE(16:0)-H | LPE | C21 H43 O7 N1 P1 | 19,145258 | 452,27827 | 1,34E+06 |

**Table S4.**Significant Lipid Results for Pork Meatballs

| **LipidIon** | **Class** | | **Formula** | **Rt** | **CalcMz** | **X Area** |
| --- | --- | --- | --- | --- | --- | --- |
| LPC(15:0)+H | | LPC | C23 H49 O7 N1 P1 | 20,497501 | 482,32412 | 1,57E+07 |
| LPC(16:0e)+H | | LPC | C24 H53 O6 N1 P1 | 20,497501 | 482,3605 | 1,57E+07 |
| LPC(20:0)+H | | LPC | C28 H59 O7 N1 P1 | 15,430334 | 552,40237 | 1,69E+06 |
| LPC(20:2)+H | | LPC | C28 H55 O7 N1 P1 | 19,753198 | 548,37107 | 4,43E+06 |
| LPC(20:3)+H | | LPC | C28 H53 O7 N1 P1 | 19,465869 | 546,35542 | 1,71E+06 |
| LPE(16:0p)+H | | LPE | C21 H45 O6 N1 P1 | 20,211859 | 438,2979 | 4,99E+07 |
| LPE(16:1e)+H | | LPE | C21 H45 O6 N1 P1 | 20,211859 | 438,2979 | 4,99E+07 |
| LPE(18:1p)+H | | LPE | C23 H47 O6 N1 P1 | 20,987529 | 464,31355 | 2,18E+07 |
| LPE(20:3)+H | | LPE | C25 H47 O7 N1 P1 | 19,180204 | 504,30847 | 1,08E+07 |
| LPE(22:4)+H | | LPE | C27 H49 O7 N1 P1 | 20,073882 | 530,32412 | 7,82E+06 |
| PC(10:0p/23:0)+H | | PC | C28 H59 O7 N1 P1 | 15,430334 | 552,40237 | 1,69E+06 |
| PC(11:0/8:0)+H | | PC | C41 H83 O7 N1 P1 | 22,831919 | 732,59017 | 1,65E+07 |
| PC(12:0e/8:0)+H | | PC | C27 H55 O8 N1 P1 | 15,430334 | 552,36598 | 1,69E+06 |
| PC(18:1e/15:0)+H | | PC | C28 H59 O7 N1 P1 | 15,430334 | 552,40237 | 1,69E+06 |
| PC(4:0/10:0)+H | | PC | C41 H83 O7 N1 P1 | 22,831919 | 732,59017 | 1,65E+07 |
| PC(4:0/15:0)+H | | PC | C22 H45 O8 N1 P1 | 20,497501 | 482,28773 | 1,57E+07 |
| PC(4:0/15:2)+H | | PC | C27 H55 O8 N1 P1 | 15,430334 | 552,36598 | 1,69E+06 |
| PC(6:0/13:0)+H | | PC | C27 H51 O8 N1 P1 | 19,753198 | 548,33468 | 4,43E+06 |
| PC(8:0/24:1)+H | | PC | C27 H55 O8 N1 P1 | 15,430334 | 552,36598 | 1,69E+06 |
| PC(8:0/24:1)+H | | PC | C40 H79 O8 N1 P1 | 22,831919 | 732,55378 | 1,65E+07 |
| PC(8:0e/12:0)+H | | PC | C28 H59 O7 N1 P1 | 15,430334 | 552,40237 | 1,69E+06 |
| PC(9:0/10:0)+H | | PC | C27 H55 O8 N1 P1 | 15,430334 | 552,36598 | 1,69E+06 |
| PE(10:0e/8:0)+H | | PE | C23 H49 O7 N1 P1 | 22,071016 | 482,32412 | 1,05E+07 |
| PE(10:0p/8:0)+H | | PE | C23 H47 O7 N1 P1 | 19,644085 | 480,30847 | 1,00E+07 |
| PE(4:0/11:0)+H | | PE | C20 H41 O8 N1 P1 | 19,502102 | 454,25643 | 1,98E+06 |
| PE(4:0/13:0)+H | | PE | C22 H45 O8 N1 P1 | 22,071016 | 482,28773 | 1,05E+07 |
| PE(6:0/11:0)+H | | PE | C22 H45 O8 N1 P1 | 22,071016 | 482,28773 | 1,05E+07 |
| PE(6:0/9:0)+H | | PE | C20 H41 O8 N1 P1 | 19,502102 | 454,25643 | 1,98E+06 |
| PE(8:0/9:0)+H | | PE | C22 H45 O8 N1 P1 | 22,071016 | 482,28773 | 1,05E+07 |
| PE(8:0e/10:0)+H | | PE | C23 H49 O7 N1 P1 | 22,071016 | 482,32412 | 1,05E+07 |
| PE(8:0e/8:0)+H | | PE | C21 H45 O7 N1 P1 | 19,502102 | 454,29282 | 1,98E+06 |
| LPE(15:1)-H | | LPE | C20 H39 O7 N1 P1 | 20,194888 | 436,24697 | 3,066E+07 |
| LPE(18:2)-H | | LPE | C23 H43 O7 N1 P1 | 18,656328 | 476,27827 | 1,979E+07 |
| LPE(17:2)-H | | LPE | C22 H41 O7 N1 P1 | 20,796106 | 462,26262 | 1,247E+07 |
| LPE(17:1)-H | | LPE | C22 H43 O7 N1 P1 | 22,921233 | 464,27827 | 6,354E+06 |
| LPE(18:0)-H | | LPE | C23 H47 O7 N1 P1 | 22,052103 | 480,30957 | 5,791E+06 |
| LPE(18:1)-H | | LPE | C23 H45 O7 N1 P1 | 20,090688 | 478,29392 | 3,597E+06 |
| PA(31:3/17:3)-H | | PA | C51 H88 O8 N0 P1 | 23,890756 | 859,62223 | 3,018E+06 |
| LPE(16:0)-H | | LPE | C21 H43 O7 N1 P1 | 19,090412 | 452,27827 | 1,175E+06 |

**Table S5.** Significant Lipid Result for Pork-Beef Meatball

| **LipidIon** | **Class** | **Formula** | **Rt** | **CalcMz** | **X Area** |
| --- | --- | --- | --- | --- | --- |
| LPE(16:0p)+H | LPE | C21 H45 O6 N1 P1 | 20,223731 | 438,2979 | 4,23E+07 |
| LPE(16:1e)+H | LPE | C21 H45 O6 N1 P1 | 20,223731 | 438,2979 | 4,23E+07 |
| LPE(18:0)+H | LPE | C23 H49 O7 N1 P1 | 22,083217 | 482,32412 | 1,28E+07 |
| LPE(18:1p)+H | LPE | C23 H47 O6 N1 P1 | 20,828903 | 464,31355 | 1,81E+07 |
| PC(4:0/15:2)+H | PC | C25 H49 O8 N1 P1 | 20,582257 | 522,31903 | 2,76E+07 |
| PC(6:0/11:0)+H | PC | C36 H61 O8 N1 P1 | 27,26678 | 666,41293 | 8,76E+07 |
| PC(6:0/8:0)+H | PC | C36 H61 O8 N1 P1 | 27,26678 | 666,41293 | 8,76E+07 |
| PC(8:0e/8:0)+H | PC | C37 H65 O7 N1 P1 | 27,26678 | 666,44932 | 8,76E+07 |
| PC(8:0p/23:1)+H | PC | C37 H65 O7 N1 P1 | 27,26678 | 666,44932 | 8,76E+07 |
| PE(10:0/22:2)+H | PE | C36 H61 O8 N1 P1 | 27,444369 | 666,41293 | 8,76E+07 |
| PE(10:0e/16:2)+H | PE | C28 H57 O7 N1 P1 | 30,616119 | 550,38672 | 1,48E+09 |
| PE(10:0e/19:0)+H | PE | C31 H61 O7 N1 P1 | 22,050879 | 590,41802 | 1,16E+07 |
| PE(10:0e/22:3)+H | PE | C35 H67 O7 N1 P1 | 22,786597 | 644,46497 | 2,71E+07 |
| PE(10:0e/23:6)+H | PE | C37 H65 O7 N1 P1 | 27,444369 | 666,44932 | 8,76E+07 |
| PE(10:0p/16:1)+H | PE | C28 H57 O7 N1 P1 | 30,616119 | 550,38672 | 1,48E+09 |
| PE(10:0p/17:3)+H | PE | C31 H61 O7 N1 P1 | 22,050879 | 590,41802 | 1,16E+07 |
| PE(10:0p/19:0)+H | PE | C32 H59 O7 N1 P1 | 23,097554 | 600,40237 | 4,20E+07 |
| PE(10:0p/22:2)+H | PE | C35 H61 O7 N1 P1 | 27,69383 | 638,41802 | 4,31E+07 |
| PE(10:0p/23:5)+H | PE | C37 H65 O7 N1 P1 | 27,444369 | 666,44932 | 8,76E+07 |
| PE(11:0/17:0)+H | PE | C24 H49 O7 N1 P1 | 29,637039 | 494,32412 | 5,19E+08 |
| PE(11:0/20:3)+H | PE | C34 H63 O8 N1 P1 | 22,786597 | 644,42858 | 2,71E+07 |
| PE(12:0/17:3)+H | PE | C25 H51 O8 N1 P1 | 22,712503 | 524,33468 | 2,61E+06 |
| PE(12:0/20:2)+H | PE | C34 H63 O8 N1 P1 | 22,786597 | 644,42858 | 2,71E+07 |
| PE(12:0e/20:3)+H | PE | C35 H67 O7 N1 P1 | 22,786597 | 644,46497 | 2,71E+07 |
| PE(12:0p/14:1)+H | PE | C28 H57 O7 N1 P1 | 30,616119 | 550,38672 | 1,48E+09 |
| PE(12:0p/20:2)+H | PE | C35 H67 O7 N1 P1 | 22,786597 | 644,46497 | 2,71E+07 |
| PE(12:0p/21:5)+H | PE | C37 H65 O7 N1 P1 | 27,444369 | 666,44932 | 8,76E+07 |
| PE(13:0/21:6)+H | PE | C26 H53 O7 N1 P1 | 26,634562 | 522,35542 | 1,92E+09 |
| PE(14:0/20:2)+H | PE | C37 H71 O8 N1 P1 | 22,714234 | 688,49118 | 1,89E+07 |
| PE(14:0p/18:2)+H | PE | C35 H67 O7 N1 P1 | 22,786597 | 644,46497 | 2,71E+07 |
| PE(14:1/17:2)+H | PE | C28 H57 O7 N1 P1 | 30,616119 | 550,38672 | 1,48E+09 |
| PE(15:0/19:2)+H | PE | C38 H73 O8 N1 P1 | 23,873171 | 702,50683 | 1,41E+07 |
| PE(15:1/19:1)+H | PE | C38 H73 O8 N1 P1 | 23,873171 | 702,50683 | 1,41E+07 |
| PE(16:0/17:2)+H | PE | C37 H71 O8 N1 P1 | 22,714234 | 688,49118 | 1,89E+07 |
| PE(16:0e/23:0)+H | PE | C39 H77 O7 N1 P1 | 23,873171 | 702,54322 | 1,41E+07 |
| PE(16:1/18:1)+H | PE | C37 H71 O8 N1 P1 | 22,714234 | 688,49118 | 1,89E+07 |
| PE(16:1p/16:1)+H | PE | C35 H67 O7 N1 P1 | 22,786597 | 644,46497 | 2,71E+07 |
| PE(16:1p/18:0)+H | PE | C38 H75 O7 N1 P1 | 22,714234 | 688,52757 | 1,89E+07 |
| PE(16:1p/19:0)+H | PE | C39 H77 O7 N1 P1 | 23,873171 | 702,54322 | 1,41E+07 |
| PE(16:2/17:0)+H | PE | C34 H63 O8 N1 P1 | 22,786597 | 644,42858 | 2,71E+07 |
| PE(17:0/21:0)+H | PE | C38 H73 O8 N1 P1 | 23,873171 | 702,50683 | 1,41E+07 |
| PE(17:1/21:5)+H | PE | C38 H73 O8 N1 P1 | 23,873171 | 702,50683 | 1,41E+07 |
| PE(18:0/10:0)+H | PE | C31 H55 O8 N1 P1 | 23,097554 | 600,36598 | 4,20E+07 |
| PE(18:0e/21:0)+H | PE | C39 H77 O7 N1 P1 | 23,873171 | 702,54322 | 1,41E+07 |
| PE(18:0p/21:5)+H | PE | C39 H77 O7 N1 P1 | 23,873171 | 702,54322 | 1,41E+07 |
| PE(18:1/20:5)+H | PE | C37 H71 O8 N1 P1 | 22,714234 | 688,49118 | 1,89E+07 |
| PE(18:1p/16:0)+H | PE | C38 H75 O7 N1 P1 | 22,714234 | 688,52757 | 1,89E+07 |
| PE(18:1p/8:0)+H | PE | C45 H79 O7 N1 P1 | 22,540155 | 776,55887 | 2,50E+06 |
| PE(18:2/20:4)+H | PE | C36 H67 O8 N1 P1 | 25,801221 | 672,45988 | 1,12E+07 |
| PE(18:2e/16:0)+H | PE | C38 H75 O7 N1 P1 | 22,714234 | 688,52757 | 1,89E+07 |
| PE(18:2e/8:0)+H | PE | C45 H79 O7 N1 P1 | 22,540155 | 776,55887 | 2,50E+06 |
| PE(18:2p/14:0)+H | PE | C35 H67 O7 N1 P1 | 22,786597 | 644,46497 | 2,71E+07 |
| PE(18:4/8:0)+H | PE | C47 H83 O8 N1 P1 | 22,454605 | 820,58508 | 2,78E+06 |
| PE(19:0/19:0)+H | PE | C31 H55 O8 N1 P1 | 23,097554 | 600,36598 | 4,20E+07 |
| PE(19:1/22:0)+H | PE | C37 H71 O8 N1 P1 | 22,714234 | 688,49118 | 1,89E+07 |
| PE(20:1/13:1)+H | PE | C38 H75 O7 N1 P1 | 22,714234 | 688,52757 | 1,89E+07 |
| PE(20:1/14:1)+H | PE | C38 H73 O8 N1 P1 | 23,873171 | 702,50683 | 1,41E+07 |
| PE(4:0/13:0)+H | PE | C21 H43 O8 N1 P1 | 23,169672 | 468,27208 | 8,00E+07 |
| PE(4:0/16:0)+H | PE | C23 H45 O8 N1 P1 | 29,637039 | 494,28773 | 5,19E+08 |
| PE(4:0/19:5)+H | PE | C27 H49 O8 N1 P1 | 22,119927 | 546,31903 | 1,48E+07 |
| PE(4:0/20:5)+H | PE | C28 H47 O8 N1 P1 | 23,167972 | 556,30338 | 5,78E+07 |
| PE(4:0/22:5)+H | PE | C31 H55 O8 N1 P1 | 23,097554 | 600,36598 | 4,20E+07 |
| PE(4:0/24:0)+H | PE | C31 H53 O8 N1 P1 | 25,141354 | 598,35033 | 5,28E+06 |
| PE(6:0/11:0)+H | PE | C21 H43 O8 N1 P1 | 23,169672 | 468,27208 | 8,00E+07 |
| PE(6:0/19:2)+H | PE | C27 H53 O8 N1 P1 | 30,616119 | 550,35033 | 1,48E+09 |
| PE(6:0/20:5)+H | PE | C31 H55 O8 N1 P1 | 23,097554 | 600,36598 | 4,20E+07 |
| PE(6:0/22:0)+H | PE | C31 H53 O8 N1 P1 | 25,141354 | 598,35033 | 5,28E+06 |
| PE(6:0/9:0)+H | PE | C34 H57 O8 N1 P1 | 27,69383 | 638,38163 | 4,31E+07 |
| PE(8:0/10:0)+H | PE | C20 H41 O8 N1 P1 | 19,518632 | 454,25643 | 1,39E+06 |
| PE(8:0/12:0)+H | PE | C23 H47 O8 N1 P1 | 19,765149 | 496,30338 | 3,15E+07 |
| PE(8:0/24:2)+H | PE | C36 H61 O8 N1 P1 | 27,444369 | 666,41293 | 8,76E+07 |
| PE(8:0/9:0)+H | PE | C21 H43 O8 N1 P1 | 23,169672 | 468,27208 | 8,00E+07 |
| PE(8:0e/13:0)+H | PE | C24 H51 O7 N1 P1 | 26,151847 | 496,33977 | 2,83E+07 |
| PE(8:0e/18:2)+H | PE | C28 H57 O7 N1 P1 | 30,616119 | 550,38672 | 1,48E+09 |
| PE(8:0e/19:4)+H | PE | C31 H61 O7 N1 P1 | 22,050879 | 590,41802 | 1,16E+07 |
| PE(8:0e/19:5)+H | PE | C32 H59 O7 N1 P1 | 23,097554 | 600,40237 | 4,20E+07 |
| PE(8:0e/21:0)+H | PE | C32 H57 O7 N1 P1 | 25,141354 | 598,38672 | 5,28E+06 |
| PE(8:0e/8:0)+H | PE | C37 H65 O7 N1 P1 | 27,444369 | 666,44932 | 8,76E+07 |
| PE(8:0p/10:0)+H | PE | C22 H47 O7 N1 P1 | 23,169672 | 468,30847 | 8,00E+07 |
| PE(8:0p/15:2)+H | PE | C28 H57 O7 N1 P1 | 29,669785 | 550,38672 | 1,49E+09 |
| PE(8:0p/18:1)+H | PE | C28 H53 O7 N1 P1 | 22,119927 | 546,35542 | 1,48E+07 |
| PE(8:0p/19:3)+H | PE | C31 H61 O7 N1 P1 | 22,050879 | 590,41802 | 1,16E+07 |
| PE(8:0p/19:4)+H | PE | C32 H59 O7 N1 P1 | 23,097554 | 600,40237 | 4,20E+07 |
| PE(8:0p/22:6)+H | PE | C35 H61 O7 N1 P1 | 27,69383 | 638,41802 | 4,31E+07 |
| PE(9:0/16:2)+H | PE | C27 H53 O8 N1 P1 | 30,616119 | 550,35033 | 1,48E+09 |
| PE(9:0/23:6)+H | PE | C36 H61 O8 N1 P1 | 27,444369 | 666,41293 | 8,76E+07 |
| PG(4:0/21:1)+H | PG | C30 H50 O10 N0 P1 | 29,072929 | 601,31361 | 3,65E+06 |
| PG(8:0/20:4)+H | PG | C31 H56 O10 N0 P1 | 26,712525 | 619,36056 | 1,05E+08 |
| PG(8:0p/24:7)+H | PG | C38 H72 O9 N0 P1 | 26,639633 | 703,49085 | 6,81E+06 |
| PI(19:1/19:4)+H | PI | C46 H80 O13 N0 P1 | 26,536314 | 871,53311 | 2,96E+08 |
| PI(24:0/22:5)+H | PI | C47 H84 O12 N0 P1 | 30,550482 | 871,56949 | 2,96E+08 |
| PS(6:0/19:0)+H | PS | C34 H53 O10 N1 P1 | 28,071095 | 666,34016 | 8,76E+07 |
| LPE(15:1)-H | LPE | C20 H39 O7 N1 P1 | 20,207637 | 436,24697 | 2,54E+07 |
| LPE(17:2)-H | LPE | C22 H41 O7 N1 P1 | 20,809842 | 462,26262 | 1,03E+07 |

**Table S6.** Lipid Significant Result for Pork-Goat Meatball

| **LipidIon** | **Class** | **Formula** | **Rt** | | **CalcMz** | **X Area** |
| --- | --- | --- | --- | --- | --- | --- |
| LPE(16:0p)+H | LPE | C21 H45 O6 N1 P1 | | 20,226484 | 438,2979 | 1,45E+07 |
| LPE(16:1e)+H | LPE | C21 H45 O6 N1 P1 | | 20,226484 | 438,2979 | 1,45E+07 |
| LPE(18:0)+H | LPE | C23 H49 O7 N1 P1 | | 22,076014 | 482,32412 | 5,02E+06 |
| LPE(18:1p)+H | LPE | C23 H47 O6 N1 P1 | | 20,890917 | 464,31355 | 5,08E+06 |
| PC(6:0/8:0)+H | PC | C36 H61 O8 N1 P1 | | 20,612442 | 482,28773 | 2,07E+06 |
| PC(8:0e/8:0)+H | PC | C37 H65 O7 N1 P1 | | 19,336525 | 496,33977 | 8,64E+06 |
| PC(8:0p/23:1)+H | PC | C37 H65 O7 N1 P1 | | 23,579781 | 702,54322 | 1,01E+07 |
| PE(10:0/22:2)+H | PE | C36 H61 O8 N1 P1 | | 22,697196 | 688,49118 | 1,05E+07 |
| PE(10:0e/16:2)+H | PE | C28 H57 O7 N1 P1 | | 22,041016 | 590,41802 | 1,19E+07 |
| PE(10:0e/22:3)+H | PE | C35 H67 O7 N1 P1 | | 25,164599 | 672,49627 | 9,62E+06 |
| PE(10:0e/23:6)+H | PE | C37 H65 O7 N1 P1 | | 30,036618 | 680,46497 | 2,98E+07 |
| PE(10:0p/16:1)+H | PE | C28 H57 O7 N1 P1 | | 22,041016 | 590,41802 | 1,19E+07 |
| PE(10:0p/17:3)+H | PE | C31 H61 O7 N1 P1 | | 23,075892 | 600,40237 | 2,24E+07 |
| PE(10:0p/19:0)+H | PE | C32 H59 O7 N1 P1 | | 21,938282 | 634,48062 | 5,62E+06 |
| PE(10:0p/22:2)+H | PE | C35 H61 O7 N1 P1 | | 25,164599 | 672,49627 | 9,62E+06 |
| PE(10:0p/23:5)+H | PE | C37 H65 O7 N1 P1 | | 30,036618 | 680,46497 | 2,98E+07 |
| PE(11:0/20:3)+H | PE | C34 H63 O8 N1 P1 | | 25,164599 | 672,45988 | 9,62E+06 |
| PE(12:0/17:3)+H | PE | C25 H51 O8 N1 P1 | | 22,803802 | 644,42858 | 1,52E+07 |
| PE(12:0/20:2)+H | PE | C34 H63 O8 N1 P1 | | 22,697196 | 688,49118 | 1,05E+07 |
| PE(12:0e/20:3)+H | PE | C35 H67 O7 N1 P1 | | 25,164599 | 672,49627 | 9,62E+06 |
| PE(12:0p/14:1)+H | PE | C28 H57 O7 N1 P1 | | 22,041016 | 590,41802 | 1,19E+07 |
| PE(12:0p/20:2)+H | PE | C35 H67 O7 N1 P1 | | 25,164599 | 672,49627 | 9,62E+06 |
| PE(12:0p/21:5)+H | PE | C37 H65 O7 N1 P1 | | 30,036618 | 680,46497 | 2,98E+07 |
| PE(13:0/21:6)+H | PE | C26 H53 O7 N1 P1 | | 30,568793 | 708,45988 | 3,05E+07 |
| PE(14:0p/18:2)+H | PE | C35 H67 O7 N1 P1 | | 25,164599 | 672,49627 | 9,62E+06 |
| PE(16:0e/23:0)+H | PE | C39 H77 O7 N1 P1 | | 22,563414 | 776,65277 | 1,50E+06 |
| PE(16:1p/16:1)+H | PE | C35 H67 O7 N1 P1 | | 25,164599 | 672,49627 | 9,62E+06 |
| PE(16:1p/18:0)+H | PE | C38 H75 O7 N1 P1 | | 23,804999 | 702,54322 | 3,51E+06 |
| PE(17:0/21:0)+H | PE | C38 H73 O8 N1 P1 | | 22,563414 | 776,61638 | 3,08E+06 |
| PE(18:0e/21:0)+H | PE | C39 H77 O7 N1 P1 | | 22,563414 | 776,65277 | 1,50E+06 |
| PE(18:1p/16:0)+H | PE | C38 H75 O7 N1 P1 | | 23,804999 | 702,54322 | 3,51E+06 |
| PE(18:1p/8:0)+H | PE | C45 H79 O7 N1 P1 | | 22,041016 | 590,41802 | 1,19E+07 |
| PE(18:2e/16:0)+H | PE | C38 H75 O7 N1 P1 | | 23,804999 | 702,54322 | 3,51E+06 |
| PE(18:2e/8:0)+H | PE | C45 H79 O7 N1 P1 | | 22,041016 | 590,41802 | 1,19E+07 |
| PE(18:2p/14:0)+H | PE | C35 H67 O7 N1 P1 | | 25,164599 | 672,49627 | 9,62E+06 |
| PE(18:4/8:0)+H | PE | C47 H83 O8 N1 P1 | | 23,075892 | 600,36598 | 2,24E+07 |
| PE(19:0/19:0)+H | PE | C31 H55 O8 N1 P1 | | 22,563414 | 776,61638 | 3,08E+06 |
| PE(20:1/13:1)+H | PE | C38 H75 O7 N1 P1 | | 23,804999 | 702,50683 | 3,51E+06 |
| PE(4:0/13:0)+H | PE | C21 H43 O8 N1 P1 | | 22,076014 | 482,28773 | 5,02E+06 |
| PE(4:0/19:5)+H | PE | C27 H49 O8 N1 P1 | | 23,181486 | 556,30338 | 3,15E+07 |
| PE(4:0/20:5)+H | PE | C28 H47 O8 N1 P1 | | 23,875808 | 570,31903 | 7,14E+06 |
| PE(4:0/22:5)+H | PE | C31 H55 O8 N1 P1 | | 25,129274 | 598,35033 | 3,50E+06 |
| PE(6:0/11:0)+H | PE | C21 H43 O8 N1 P1 | | 22,076014 | 482,28773 | 5,02E+06 |
| PE(6:0/19:2)+H | PE | C27 H53 O8 N1 P1 | | 22,041016 | 590,38163 | 1,19E+07 |
| PE(6:0/20:5)+H | PE | C31 H55 O8 N1 P1 | | 25,129274 | 598,35033 | 3,50E+06 |
| PE(6:0/9:0)+H | PE | C34 H57 O8 N1 P1 | | 19,089093 | 454,25643 | 2,05E+06 |
| PE(8:0/10:0)+H | PE | C20 H41 O8 N1 P1 | | 26,171229 | 496,30338 | 2,13E+07 |
| PE(8:0/24:2)+H | PE | C36 H61 O8 N1 P1 | | 22,697196 | 688,49118 | 1,05E+07 |
| PE(8:0/9:0)+H | PE | C21 H43 O8 N1 P1 | | 22,076014 | 482,28773 | 5,02E+06 |
| PE(8:0e/18:2)+H | PE | C28 H57 O7 N1 P1 | | 22,041016 | 590,41802 | 1,19E+07 |
| PE(8:0e/19:4)+H | PE | C31 H61 O7 N1 P1 | | 23,075892 | 600,40237 | 2,24E+07 |
| PE(8:0e/19:5)+H | PE | C32 H59 O7 N1 P1 | | 25,129274 | 598,38672 | 3,50E+06 |
| PE(8:0e/8:0)+H | PE | C37 H65 O7 N1 P1 | | 19,089093 | 454,29282 | 2,05E+06 |
| PE(8:0p/10:0)+H | PE | C22 H47 O7 N1 P1 | | 20,118482 | 480,30847 | 1,74E+07 |
| PE(8:0p/15:2)+H | PE | C28 H57 O7 N1 P1 | | 22,109681 | 546,35542 | 1,48E+07 |
| PE(8:0p/18:1)+H | PE | C28 H53 O7 N1 P1 | | 22,041016 | 590,41802 | 1,19E+07 |
| PE(8:0p/19:3)+H | PE | C31 H61 O7 N1 P1 | | 23,075892 | 600,40237 | 2,24E+07 |
| PE(8:0p/19:4)+H | PE | C32 H59 O7 N1 P1 | | 25,129274 | 598,38672 | 3,50E+06 |
| PE(9:0/16:2)+H | PE | C27 H53 O8 N1 P1 | | 22,041016 | 590,38163 | 1,19E+07 |
| PE(9:0/23:6)+H | PE | C36 H61 O8 N1 P1 | | 30,036618 | 680,42858 | 2,98E+07 |
| PG(4:0/21:1)+H | PG | C30 H50 O10 N0 P1 | | 27,704463 | 623,39186 | 3,03E+07 |
| PI(24:0/22:5)+H | PI | C47 H84 O12 N0 P1 | | 28,00502 | 997,67396 | 2,95E+06 |
| PS(6:0/19:0)+H | PS | C34 H53 O10 N1 P1 | | 27,9797 | 638,40276 | 1,10E+07 |
| LPE(15:1)-H | LPE | C20 H39 O7 N1 P1 | | 20,207943 | 436,24697 | 7,31E+06 |
| LPE(17:2)-H | LPE | C22 H41 O7 N1 P1 | | 20,808023 | 462,26262 | 1,70E+06 |

**Table S7.** Lipid Significant Result for Beef-Goat Meatball

| **LipidIon** | **Class** | **Formula** | **Rt** | **CalcMz** | **X Area** |
| --- | --- | --- | --- | --- | --- |
| LPE(16:0p)+H | LPE | C21 H45 O6 N1 P1 | 20,218789 | 438,2979 | 3,37E+06 |
| LPE(16:1e)+H | LPE | C21 H45 O6 N1 P1 | 20,218789 | 438,2979 | 3,37E+06 |
| PC(6:0/11:0)+H | PC | C36 H61 O8 N1 P1 | 22,603315 | 524,33468 | 2,68E+06 |
| PC(6:0/8:0)+H | PC | C36 H61 O8 N1 P1 | 25,02813 | 482,28773 | 2,25E+06 |
| PC(8:0e/8:0)+H | PC | C37 H65 O7 N1 P1 | 19,96782 | 496,33977 | 1,21E+07 |
| PC(8:0p/23:1)+H | PC | C37 H65 O7 N1 P1 | 23,81759 | 702,54322 | 8,84E+06 |
| PE(10:0/22:2)+H | PE | C36 H61 O8 N1 P1 | 22,704261 | 688,49118 | 9,33E+06 |
| PE(10:0e/16:2)+H | PE | C28 H57 O7 N1 P1 | 22,042454 | 590,41802 | 1,26E+07 |
| PE(10:0e/22:3)+H | PE | C35 H67 O7 N1 P1 | 25,792817 | 672,49627 | 6,63E+06 |
| PE(10:0e/23:6)+H | PE | C37 H65 O7 N1 P1 | 29,205972 | 680,46497 | 6,10E+06 |
| PE(10:0p/16:1)+H | PE | C28 H57 O7 N1 P1 | 22,042454 | 590,41802 | 1,26E+07 |
| PE(10:0p/17:3)+H | PE | C31 H61 O7 N1 P1 | 22,912938 | 600,40237 | 2,11E+07 |
| PE(10:0p/19:0)+H | PE | C32 H59 O7 N1 P1 | 22,010127 | 634,48062 | 2,90E+06 |
| PE(10:0p/22:2)+H | PE | C35 H61 O7 N1 P1 | 25,792817 | 672,49627 | 6,63E+06 |
| PE(10:0p/23:5)+H | PE | C37 H65 O7 N1 P1 | 29,205972 | 680,46497 | 6,10E+06 |
| PE(11:0/20:3)+H | PE | C34 H63 O8 N1 P1 | 25,792817 | 672,45988 | 6,63E+06 |
| PE(12:0/17:3)+H | PE | C25 H51 O8 N1 P1 | 22,775399 | 644,42858 | 1,40E+07 |
| PE(12:0/20:2)+H | PE | C34 H63 O8 N1 P1 | 22,704261 | 688,49118 | 9,33E+06 |
| PE(12:0e/20:3)+H | PE | C35 H67 O7 N1 P1 | 25,792817 | 672,49627 | 6,63E+06 |
| PE(12:0p/14:1)+H | PE | C28 H57 O7 N1 P1 | 22,042454 | 590,41802 | 1,26E+07 |
| PE(12:0p/20:2)+H | PE | C35 H67 O7 N1 P1 | 25,792817 | 672,49627 | 6,63E+06 |
| PE(12:0p/21:5)+H | PE | C37 H65 O7 N1 P1 | 29,205972 | 680,46497 | 6,10E+06 |
| PE(14:0/20:2)+H | PE | C37 H71 O8 N1 P1 | 25,685652 | 716,52248 | 3,23E+06 |
| PE(14:0p/18:2)+H | PE | C35 H67 O7 N1 P1 | 25,792817 | 672,49627 | 3,65E+06 |
| PE(15:1/19:1)+H | PE | C38 H73 O8 N1 P1 | 25,685652 | 716,52248 | 3,23E+06 |
| PE(16:1p/16:1)+H | PE | C35 H67 O7 N1 P1 | 25,792817 | 672,49627 | 6,63E+06 |
| PE(17:0/21:0)+H | PE | C38 H73 O8 N1 P1 | 22,52989 | 776,61638 | 1,67E+06 |
| PE(18:1p/8:0)+H | PE | C45 H79 O7 N1 P1 | 22,042454 | 590,41802 | 1,26E+07 |
| PE(18:2e/8:0)+H | PE | C45 H79 O7 N1 P1 | 22,042454 | 590,41802 | 1,26E+07 |
| PE(18:2p/14:0)+H | PE | C35 H67 O7 N1 P1 | 25,792817 | 672,49627 | 6,63E+06 |
| PE(18:4/8:0)+H | PE | C47 H83 O8 N1 P1 | 22,912938 | 600,36598 | 2,11E+07 |
| PE(19:0/19:0)+H | PE | C31 H55 O8 N1 P1 | 22,52989 | 776,61638 | 1,67E+06 |
| PE(4:0/13:0)+H | PE | C21 H43 O8 N1 P1 | 22,045898 | 482,28773 | 1,66E+07 |
| PE(4:0/19:5)+H | PE | C27 H49 O8 N1 P1 | 22,983795 | 556,30338 | 3,08E+07 |
| PE(4:0/20:5)+H | PE | C28 H47 O8 N1 P1 | 23,91157 | 570,31903 | 6,63E+06 |
| PE(4:0/22:5)+H | PE | C31 H55 O8 N1 P1 | 25,090295 | 598,35033 | 3,61E+06 |
| PE(6:0/11:0)+H | PE | C21 H43 O8 N1 P1 | 22,045898 | 482,28773 | 1,66E+07 |
| PE(6:0/19:2)+H | PE | C27 H53 O8 N1 P1 | 22,042454 | 590,38163 | 1,26E+07 |
| PE(6:0/20:5)+H | PE | C31 H55 O8 N1 P1 | 25,090295 | 598,35033 | 3,61E+06 |
| PE(6:0/9:0)+H | PE | C34 H57 O8 N1 P1 | 19,507182 | 454,25643 | 6,34E+05 |
| PE(8:0/10:0)+H | PE | C20 H41 O8 N1 P1 | 26,178484 | 496,30338 | 2,11E+07 |
| PE(8:0/24:2)+H | PE | C36 H61 O8 N1 P1 | 22,704261 | 688,49118 | 9,33E+06 |
| PE(8:0/9:0)+H | PE | C21 H43 O8 N1 P1 | 22,045898 | 482,28773 | 1,66E+07 |
| PE(8:0e/18:2)+H | PE | C28 H57 O7 N1 P1 | 22,042454 | 590,41802 | 1,26E+07 |
| PE(8:0e/19:4)+H | PE | C31 H61 O7 N1 P1 | 22,912938 | 600,40237 | 2,11E+07 |
| PE(8:0e/19:5)+H | PE | C32 H59 O7 N1 P1 | 25,090295 | 598,38672 | 3,61E+06 |
| PE(8:0e/8:0)+H | PE | C37 H65 O7 N1 P1 | 19,507182 | 454,29282 | 6,71E+06 |
| PE(8:0p/10:0)+H | PE | C22 H47 O7 N1 P1 | 19,684416 | 480,30847 | 1,34E+07 |
| PE(8:0p/15:2)+H | PE | C28 H57 O7 N1 P1 | 22,14685 | 546,35542 | 1,61E+07 |
| PE(8:0p/18:1)+H | PE | C28 H53 O7 N1 P1 | 22,042454 | 590,41802 | 1,26E+07 |
| PE(8:0p/19:3)+H | PE | C31 H61 O7 N1 P1 | 22,912938 | 600,40237 | 2,11E+07 |
| PE(8:0p/19:4)+H | PE | C32 H59 O7 N1 P1 | 25,090295 | 598,38672 | 3,61E+06 |
| PE(9:0/16:2)+H | PE | C27 H53 O8 N1 P1 | 22,042454 | 590,38163 | 1,26E+07 |
| PE(9:0/23:6)+H | PE | C36 H61 O8 N1 P1 | 29,205972 | 680,42858 | 6,10E+06 |
| PG(4:0/21:1)+H | PG | C30 H50 O10 N0 P1 | 27,737164 | 623,39186 | 4,76E+06 |
| PS(6:0/19:0)+H | PS | C34 H53 O10 N1 P1 | 26,668904 | 638,40276 | 2,22E+07 |
| LPE(15:1)-H | LPE | C20 H39 O7 N1 P1 | 20,20272 | 436,24697 | 1,84E+06 |

**Table S8. Total Ion Chromatogram (TIC) of Single and Combined Meatball Samples**

| **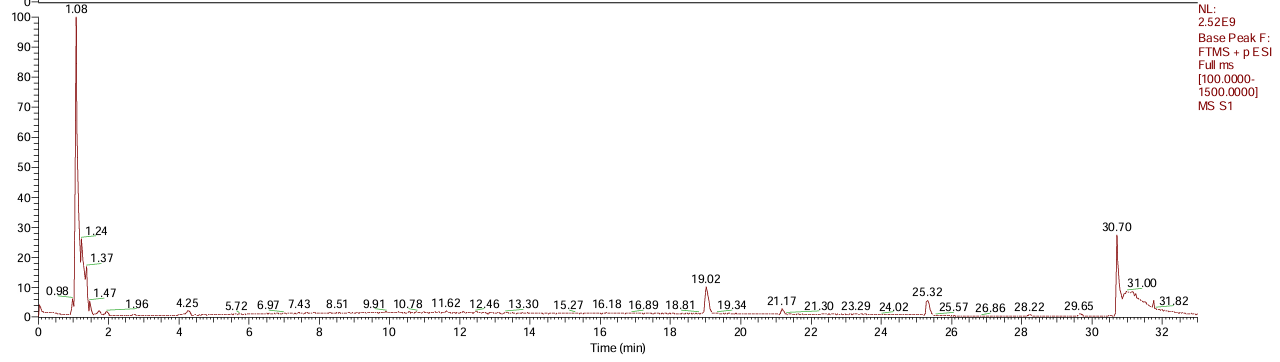**  **TIC Meatball beef in ESI+** |
| --- |
| **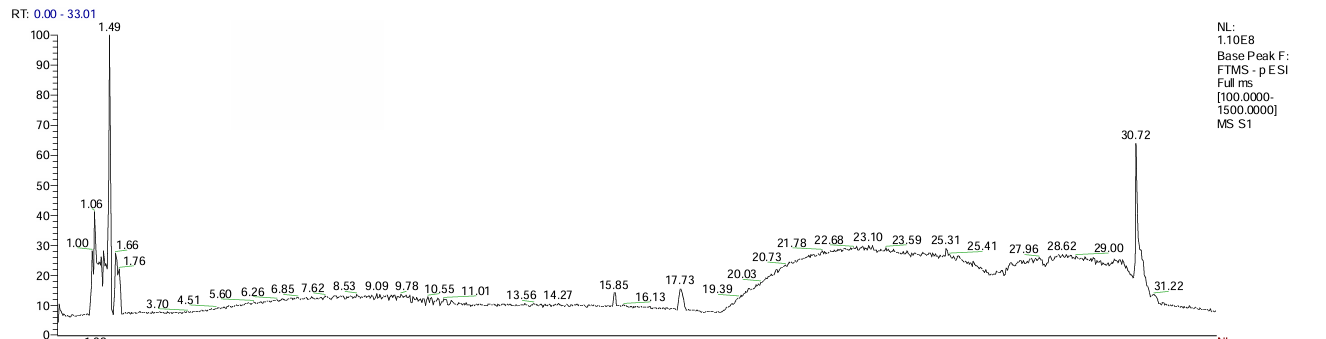**  **TIC Meatball beef in ESI-** |
| **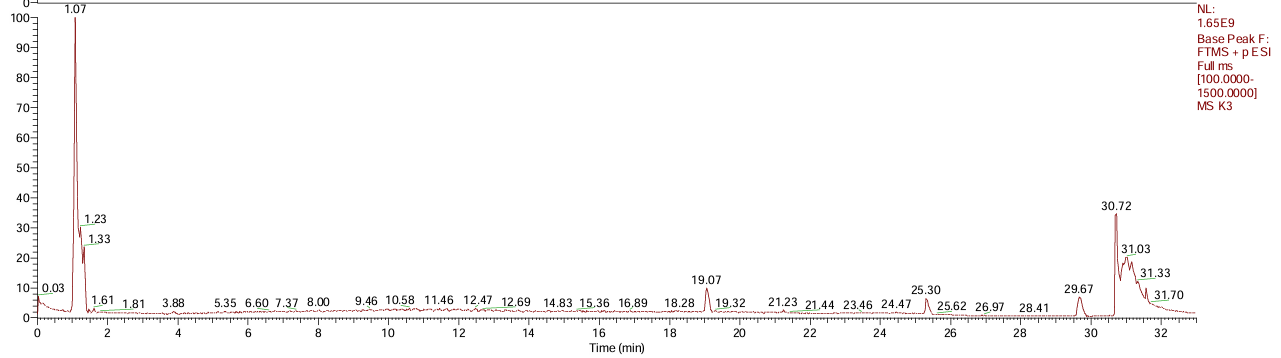**  **TIC Meatball goat in ESI+** |
| **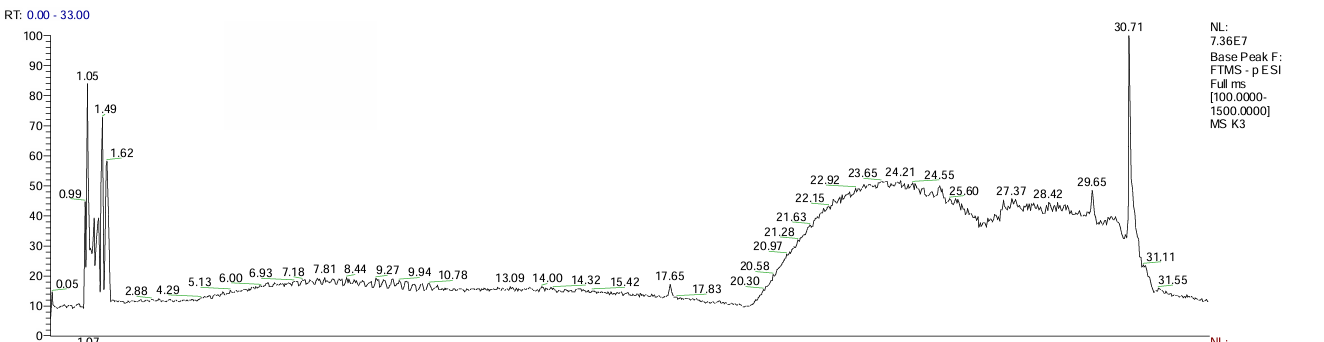**  **TIC Meatball goat in ESI-** |
| **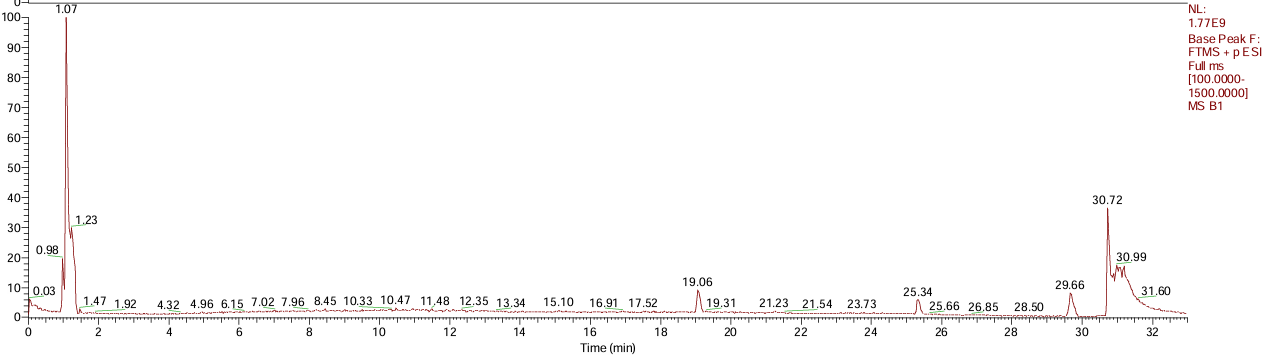**  **TIC Meatball pork in ESI+** |
| **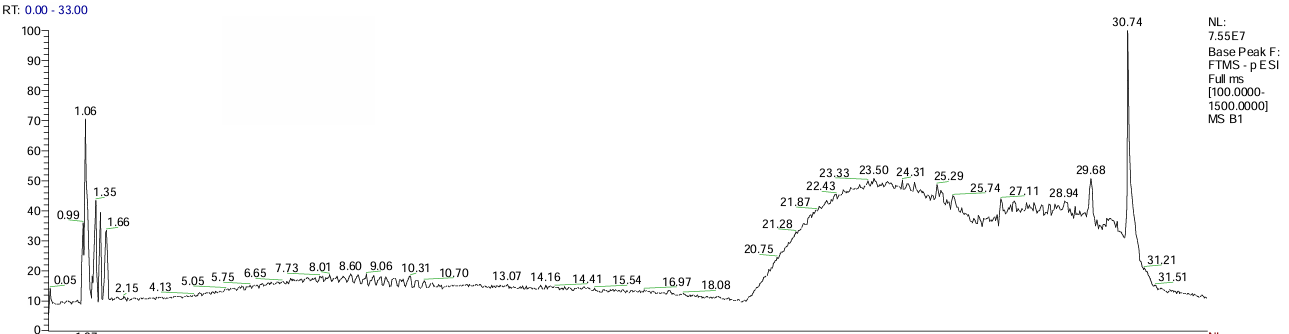**  **TIC Meatball pork in ESI-** |
| **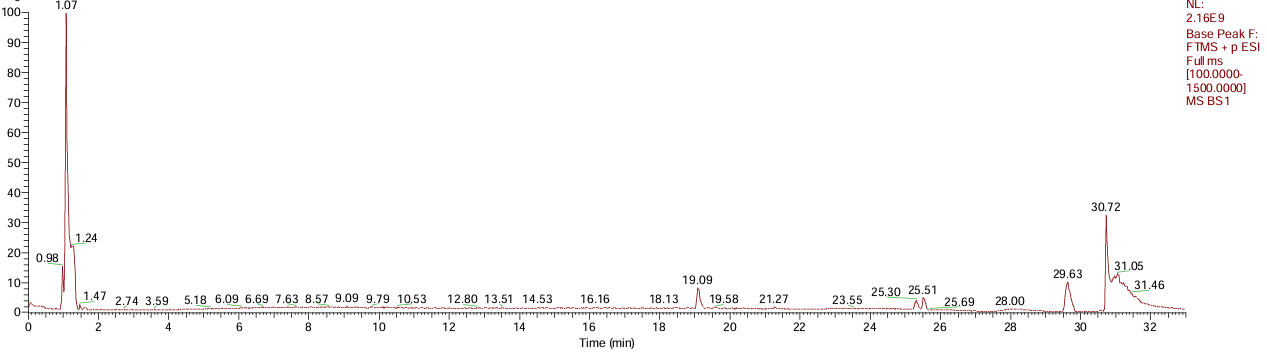**  **TIC Combined Meatball Beef and Pork in ESI+** |
| **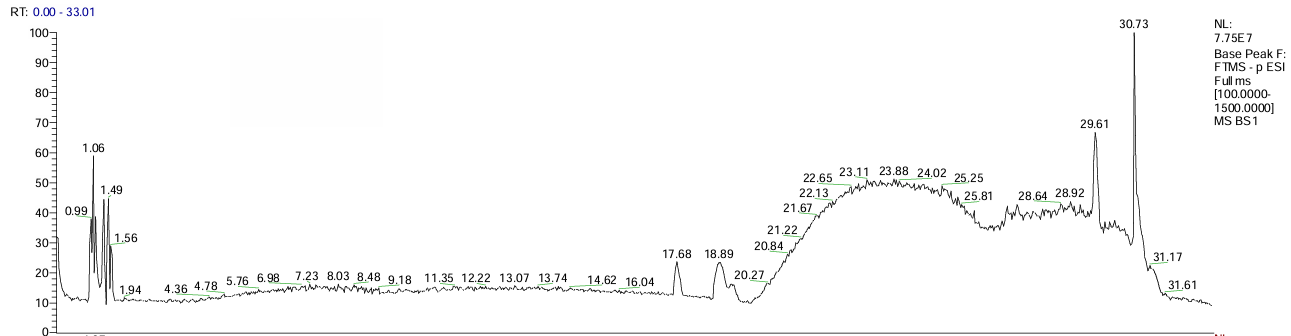**  **TIC Combined Meatball Beef and Pork in ESI-** |
| **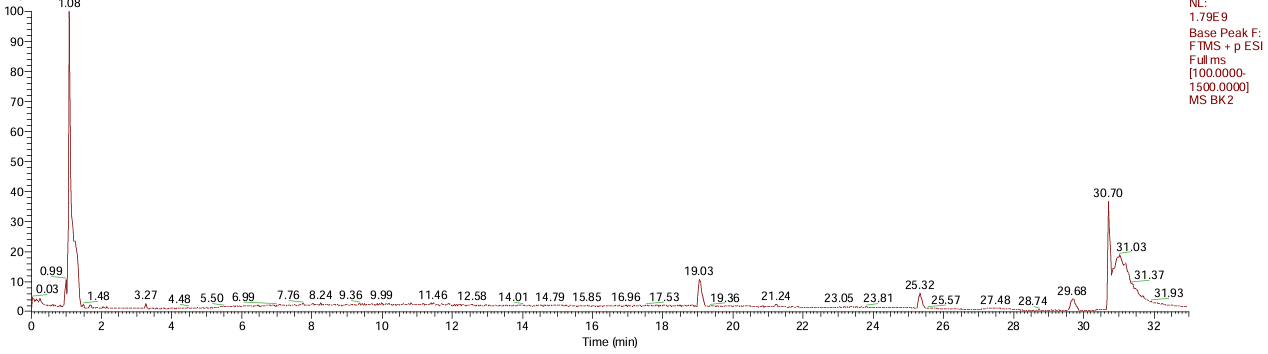**  **TIC Combined Meatball Goat and Pork in ESI+** |
| **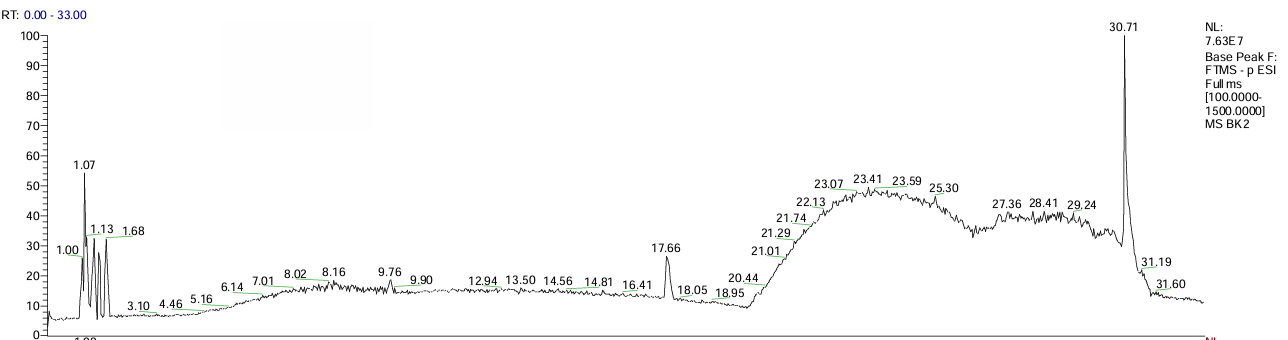**  **TIC Combined Meatball Goat and Pork in ESI-** |
| **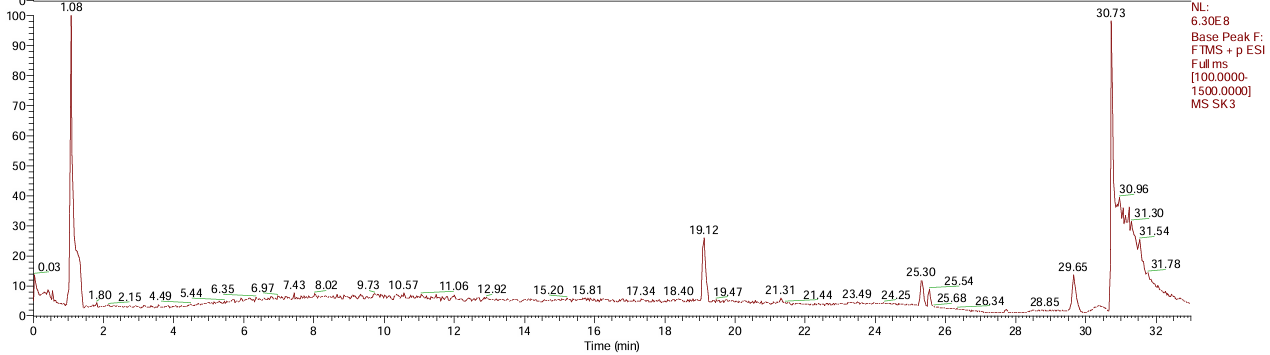**  **TIC Combined Meatball Beef and Goat in ESI+** |
| **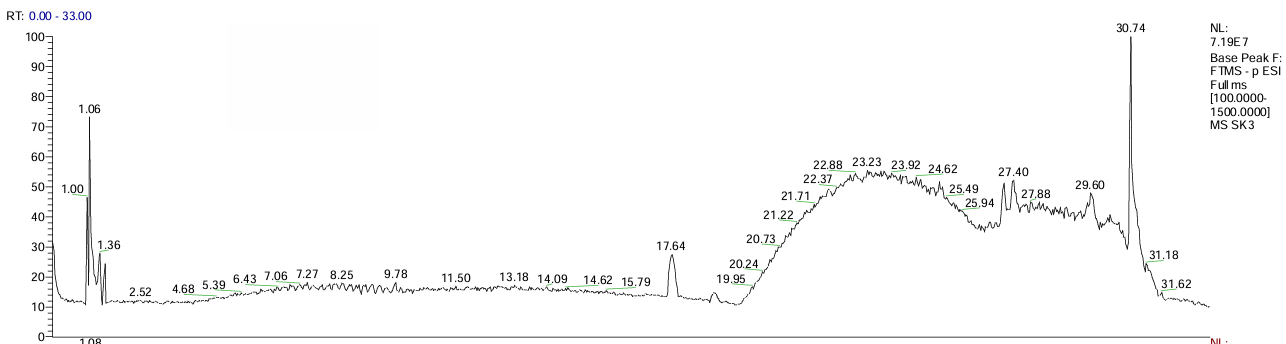**  **TIC Combined Meatball Beef and Goat in ESI-** |

**Table S9. MS Conditions for UHPLC-HRMS**

| **Full MS** | | |
| --- | --- | --- |
| Resolution | : | 70,000 |
| AGC Target | : | 3e6 |
| Maximum IT | : | 100 ms |
| Scan Range | : | 100 to 1500 m/z |
| **dd-MS*/dd-SIM** | | |
| Resolution | : | 17,500 |
| AGC Target | : | 1e5 |
| Maximum IT | : | 50 ms |
| Loop count | : | 5 |
| TopN | : | 5 |
| Isolation window | : | 4.0 m/z |
| Fixed first mass | : | - |
| (N)CE/stepped (N)CE nce | : | 18, 35, 53 |
| **dd Settings** | | |
| Minimum AGC Target | : | 8,00e3 |
| Intensity thereshold | : | 1.6e5 |
| Apex trigger | : | - |
| Charge exclusion | : | - |
| Peptide match | : | Prefered |
| Exclude isotopes | : | On |
| Dynamic exclusion | : | 10.0 s |

| **HESI Source** | | |
| --- | --- | --- |
| **Actual** | | |
| Sheath gas flow rate | 15 | 0 |
| Aux gas flow rate | 3 | 0 |
| Sweep gas flow rate | 0 | 0 |
| Spray voltage ([kV]) | 3.80 | 0.00 |
| Spray current (µA) |  | 0.10 |
| Capillary temp (°C) | 320 | 320 |
| S-lens RF Level | 50.0 |  |
| Aux gas heater temp (°C) | 0 | 39 |
